# Effects of Nurse‐Led Interventions on Adherence and Clinical Outcomes Among Adults Receiving Noninvasive Ventilation: A Systematic Review of Randomized Controlled Trials

**DOI:** 10.1155/nrp/7876955

**Published:** 2026-10-12

**Authors:** Zhenxiu Yu, Donwiwat Saensom, Xiaomei Chang, Meiqi Luo, Shenglin Hu, Lijuan Xiong

**Affiliations:** ^1^ Department of ICU Ya’an People’s Hospital Ya’an China yasyy.com; ^2^ Faculty of Nursing Khon Kaen University Khon Kaen Thailand kku.ac.th; ^3^ Department of Anorectal Ya’an People’s Hospital Ya’an China yasyy.com; ^4^ Department of Nursing Ya’an People’s Hospital Ya’an China yasyy.com; ^5^ Department of Otolaryngology–Head and Neck Surgery Ya’an People’s Hospital Ya’an China yasyy.com

**Keywords:** adherence, adults, clinical outcome, noninvasive ventilation, nurse-led, obstructive sleep apnea, quality of life, self-efficacy, sleep

## Abstract

**Background:**

Noninvasive ventilation (NIV) is a cornerstone therapy for respiratory diseases, but its effectiveness depends on patient adherence, which remains suboptimal. Although nurses play a key role in NIV management, the effects of nurse‐led interventions on adherence and clinical outcomes remain unclear.

**Objective:**

To evaluate the effects of nurse‐led interventions on NIV adherence and clinical outcomes in adults.

**Methods:**

Six databases were searched from inception to January 2026 for English randomized controlled trials (RCTs) involving adults receiving NIV. Two reviewers independently conducted study selection and data extraction. Risk of bias was assessed using the Risk of Bias 2 tool, and the findings were synthesized without meta‐analysis.

**Results:**

Sixteen RCTs involving 1757 participants were included. Risk of bias ranged from low to high across studies. Interventions included health education, motivational and behavioral strategies, and follow‐up support delivered face‐to‐face, by telephone, or via telehealth. Nurse‐led interventions may improve NIV adherence, although the effects varied across intervention types and follow‐up periods. Some studies reported improvements in symptom burden, emotional status, and selected health service outcomes, whereas findings on quality of life, self‐efficacy, and healthcare utilization were inconsistent.

**Conclusions:**

Nurse‐led interventions may improve NIV adherence, particularly when combining education with motivational and behavioral strategies and structured follow‐up.

## 1. Introduction

Noninvasive ventilation (NIV) is a cornerstone therapy for adults with obstructive sleep apnea (OSA), chronic hypercapnic respiratory failure (CHRF), and chronic obstructive pulmonary disease (COPD) and has been shown to improve respiratory status and cardiopulmonary function [1–3]. However, the clinical benefits of NIV depend largely on patient adherence, which is defined as the extent to which an individual’s behavior corresponds with the recommendations agreed upon with a healthcare provider [4]. Unfortunately, adherence to NIV is frequently suboptimal [5–7]. It is influenced by multiple interacting factors, including physiological and psychological status, health beliefs, cognitive understanding, and social support. These factors operate dynamically through triggering, facilitating, and maintaining mechanisms [8, 9].

In recent years, interventions targeting NIV adherence have included pharmacological approaches, educational programs, technical support, motivational counseling, telemonitoring, and social support [10–15]. It is widely recognized that nurses are the healthcare professionals who maintain the most frequent and sustained contact with patients during NIV therapy and play a pivotal role in treatment and care [16]. Although previous reviews have examined psychosocial and behavioral strategies to improve NIV adherence, most have focused on physician‐led or technology‐based interventions and have predominantly targeted continuous positive airway pressure (CPAP), with limited attention to nurse‐led interventions. Moreover, clinical outcomes other than adherence, including symptom control and healthcare utilization, have not been systematically evaluated.

Therefore, this review aims to systematically synthesize evidence on nurse‐led interventions for adults receiving NIV in order to clarify their effects on adherence and clinical outcomes. Specifically, this review addresses two questions: (1) What types of nurse‐led interventions have been implemented to improve NIV adherence in adult patients? (2) What are the effects of nurse‐led interventions on NIV adherence and clinical outcomes in adult patients?

## 2. Methods

### 2.1. Registration

This systematic review was registered in PROSPERO (registration number: CRD420261282553).

### 2.2. Eligibility Criteria

Quantitative studies published in English from database inception to January 2026 were eligible for inclusion. Searches across all databases were completed on January 31, 2026. Studies were included if they focused on nurse‐led management of adherence to NIV. The full inclusion criteria according to the PICO framework are presented below:

#### 2.2.1. P (Population)

Inclusion—adults (≥ 18 years) diagnosed as requiring NIV, including but not limited to OSA, CHRF, hypoxemic respiratory failure (HRF), and COPD.

Exclusion—patients with impaired consciousness.

#### 2.2.2. I (Intervention)

Inclusion—nurse‐led interventions (or interventions delivered or organized by nursing staff), including but not limited to health or patient education, comfort measures, positioning, behavioral support, and psychological support.

Exclusion—physician‐led interventions.

#### 2.2.3. C (Control)

Inclusion—standard or usual care. Studies comparing different nursing intervention strategies were also eligible.

#### 2.2.4. O (Outcome)

Inclusion—adherence (reported as duration, percentage, or based on a specific operational definition) and/or other clinical outcomes, including but not limited to quality of life (QoL), sleep‐related symptoms (e.g., daytime sleepiness and sleep quality), self‐efficacy, and health service and healthcare utilization outcomes (e.g., emergency department [ED] visits, hospital admissions, and follow‐up visits).

Exclusion—studies that did not report adherence‐related data.

#### 2.2.5. Study Characteristics

Inclusion—peer‐reviewed randomized controlled trial (RCTs) published in English from database inception to January 2026.

Exclusion—editorials, conference abstracts, opinion pieces, protocols, case reports, reviews, commentaries, and retracted articles.

### 2.3. Search Strategy

Six electronic databases were searched: CINAHL, Cochrane Library, Embase, PubMed, Scopus, and Web of Science. Reference lists of studies with full‐text reports were also screened manually. The search strategy was developed and refined collaboratively by the research team and a librarian at Khon Kaen University. Both Medical Subject Headings (MeSH) terms and free‐text terms were used. The MeSH terms included: “Adult”[Mesh], “Young Adult”[Mesh], “Aged”[Mesh], “Middle Aged”[Mesh], “Aged, 80 and over”[Mesh], “Noninvasive Ventilation”[Mesh], “Continuous Positive Airway Pressure”[Mesh], “Psychosocial Intervention”[Mesh], “Education”[Mesh], “education [Subheading]”[Mesh], “Patient Education as Topic”[Mesh], “Health Education”[Mesh], “Patient Comfort”[Mesh], “Information Motivation Behavioral Skills Model”[Mesh], “Patient Positioning”[Mesh], “Resilience, Psychological”[Mesh], “Feedback, Psychological”[Mesh], “Psychological Well‐Being”[Mesh], “Psychosocial Support Systems”[Mesh], “Treatment Adherence and Compliance”[Mesh], “Patient Compliance”[Mesh], and “Guideline Adherence”[Mesh]. The detailed search strategies for all databases are provided in Supporting File 1.

### 2.4. Study Selection Process

All retrieved records were downloaded into EndNote 21 for duplicate removal [17] and then imported into Rayyan [18]. Study selection was conducted in two stages: (1) screening of titles and abstracts and (2) full‐text review. Screening was performed independently and in a blinded manner by two reviewers. A pilot test was performed to ensure consistency, and disagreements were resolved through discussion or consultation with a third reviewer. Studies with insufficient information at the title and abstract stage were advanced to full‐text review. The study selection process is summarized in a PRISMA flow diagram [19].

### 2.5. Data Extraction Process and Data Synthesis

A standardized data extraction form was developed to collect the following information: study characteristics, participant characteristics, interventions, and outcomes. A pilot data extraction was conducted to ensure consistency, and the form was refined accordingly to enhance usability. Data extraction was then performed independently by two reviewers, with discrepancies resolved by a third reviewer. Due to heterogeneity in reporting, the findings were synthesized without meta‐analysis.

### 2.6. Quality Appraisal

Two reviewers independently assessed the risk of bias of the included studies using the Risk of Bias 2 tool [20]. This tool evaluates five domains: the randomization process, deviations from intended interventions, missing outcome data, measurement of outcomes, and selection of the reported results. Each domain was rated as low risk, some concerns, or high risk.

## 3. Results

A total of 1620 records were identified through database searching, and an additional 5 records were identified through citation searching. After removal of duplicates, 1051 unique records were screened, of which 30 were assessed for full‐text eligibility. Ultimately, 16 RCTs were included (see Figure 1).

**FIGURE 1:**
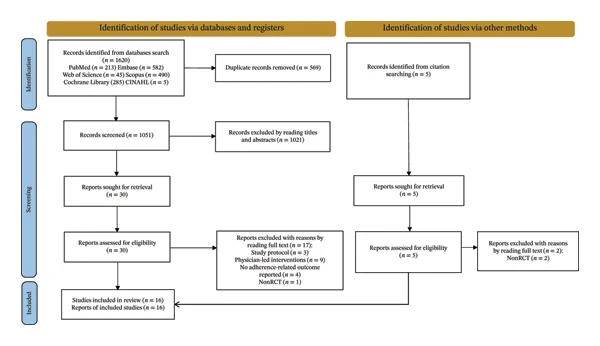
PRISMA 2020 flow diagram for studies’ selection process.

### 3.1. Study Characteristics

The included studies were published between 1999 and 2025 and were conducted across seven countries, with most undertaken in the United States (*n* = 7) and China (*n* = 4). Sample sizes ranged from 29 to 263 participants. The majority of studies enrolled patients with OSA. Among studies reporting mean age, participant ages ranged from 47.0 to 70.05 years. Most studies enrolled predominantly male participants. Among studies reporting mean BMI, the majority reported a mean BMI ≥ 30 kg/m^2^. Follow‐up periods ranged from 30 days to 12 months. Detailed characteristics of the included studies are presented in Table 1.

**TABLE 1:** Characteristics of included studies.

Study (author, year)	Study design	Country and settings	Population	Sample size (IG/CG)	NIV mode	Participant characteristics (IG/CG)	Follow‐up duration
Srikajon 2025	RCT	Siriraj Sleep Center, Faculty of Medicine, Siriraj Hospital, Bangkok, Thailand	OSA	66/66	CPAP	Age: 51.7 (13.66)/50.2 (16.26) Male: 41 (62.1)/40 (60.6) BMI: 28.7 (18.7, 59)/29.3 (17.7, 67.7) AHI: 49.4 (5.1–166.2)/42.2 (7.3–146.3) ESS: 8 (0, 23)/9 (2, 18)	3 months

Aloia 2013	RCT	Sleep Disorders Center, Lifespan Hospitals (affiliated with Brown University), Providence, Rhode Island, United States	OSA	73/80/74	PAP	Age: 51.7 (10.0)/47.0 (11.4)/52.4 (11.8) Male: 45 (62)/48 (60)/57 (77) BMI: 35.1 (7.3)/35.0 (7.3)/35.8 (8.4) AHI: 45.7 (23.8)/46.1 (23.2)/48.2 (26.2) ESS: 11.6 (5.2)/12.6 (4.9)/11.9 (5.1)	12 months

Hoy 1999	RCT	Sleep Center, Royal Infirmary, Edinburgh, United Kingdom	SAHS	40/40	CPAP	Overall age: 51 (11) Overall male: 78 (97.5) BMI: 32 (6.3)/33 (6.3) Overall AHI: 58 (31.6)/59 (31.6) Overall ESS: 13 (6.3)/13 (6.3)	6 months

Khan 2022	RCT	One pulmonary clinic, one sleep laboratory, and three clinics, Michigan, United States	OSA	28/32	PAP	Age: 58 (11.75)/50 (12.92) Male: 12 (43)/11 (34) BMI: 35.5 (7.2)/39.4 (11.5) AHI: 26.8 (25.0)/25.5 (21.6) ESS: 9.1 (0.9)/10.2 (0.9)	6 months

Kooij 2021	RCT	Teaching hospital, Rijnstate, Arnhem, the Netherlands	OSA	70/70	CPAP	Age: 52.3 (12.4)/54.3 (11.9) Male: 58 (83)/53 (76) BMI > 30: 51 (73)/46 (66) AHI: 31.0 (22.0–46.0)/30.5 (20.0–42.0) ESS score ≤ 10: 56 (84)/49 (74)	24 weeks

Rudilla 2021	RCT	Respiratory Medicine Department, La Princesa University Hospital, Madrid, Spain	OSA	42/41	CPAP	Age: 61.35 (13.11)/57.51 (12.19) Male: 32 (76)/28 (68) BMI: NR AHI: 46.30 (18.80)/49.95 (17.85) ESS: 10.35 (4.53)/13.02 (4.29)	90 days

Miller 2024	RCT	Sleep/Pulmonary Clinic, Midwestern State, United States	OSA	15/14	CPAP	Age: 50.2 (18.0)/55.6 (13.3) Male: 10 (66.7)/5 (35.7) BMI: 38.9 (9.0)/37.0 (13.3) AHI: 23.9 (17.7)/36.5 (38.8) ESS: NR	12 weeks

Sawyer 2019	RCT	Academic Medical Center with an American Academy of Sleep Medicine–accredited Sleep Center, suburban setting, United States	OSA	30/30	Oxygen/bi‐level PAP	Age: 52.5 (11.7)/50.1 (10.5) Male: 19 (63)/23 (77) BMI: 37.0 (8.1)/39.0 (10.3) AHI: 33.9 (21.3)/38.0 (28.8) ESS: 18.7 (5.1)/20.4 (5.3)	3 months

Smith 2009	RCT	Two large sleep laboratories, Kansas, United States	OSA	55/42	CPAP	Age: 63 (6.94)/63 (7.15) Male: 35 (63.6)/22 (51.5) BMI: 18.5–29: 18.5%/20.0% 30–39: 59.0%/61.8% > 40: 22.5%/18.2% AHI: 52.3 (30.4)/47.3 (30.0) ESS: NR	6 months

Chen 2015	RCT	Three sleep disorder clinics, Zhejiang Province, China	OSA	40/40	CPAP	Age: 50.2 (10.1)/50.6 (11.2) Male: 29 (72.5)/28 (70.0) BMI: 33 (2)/32 (3) AHI: 55 (4)/54 (4) ESS: 13 (1)/13 (1)	12 months

Parthasarathy 2025	RCT	Four sleep centers, United States	OSA	131/132	CPAP	Age: 50.4 (13.1)/47.9 (12.8) Male: 66 (50.7)/70 (56.0) BMI: 33.2 (7.0)/33.4 (6.8) AHI: 30.4 (26.3)/26.3 (24.8) ESS: 11.3 (5.6)/11.7 (5.2)	6 months

Wang 2012	RCT	Xiangya Hospital, Central South University, China	OSA	38/38/38/38	CPAP	Age: 30–39: 7 (18.4)/9 (23.7)/8 (21.1)/10 (26.3) 40–49: 12 (31.5)/10 (26.3)/9 (23.7)/8 (21.1) 50–59: 11 (28.9)/9 (23.7)/10 (26.3)/8 (21.1) ≥ 60: 8 (21.1)/10 (26.3)/11 (28.9)/12 (31.5) Male: 34 (89.5)/29 (76.3)/31 (81.6)/31 (81.6) BMI: 18.5–< 25: 2 (5.3)/1 (2.6)/0 (0)/2 (5.3) 25–< 30: 8 (21.1)/7 (18.4)/8 (21.1)/9 (23.7) 30–40: 21 (55.3)/20 (52.6)/21 (55.3)/19 (50.0) > 40: 7 (18.4)/10 (26.3)/9 (23.7)/8 (21.1) AHI: 35.7 (16.2)/41.2 (21.4)/43.1 (22.5)/38.5 (18.7) ESS: 13.4 (6.5)/14.7 (6.9)/14.5 (6.2)/13.9 (6.6)	12 weeks

Martinotti 2025	RCT	Sleep Center of Pneumology, Hospital of San Marino, San Marino	OSAS	30/30	CPAP	Age: 66 (10)/65 (10) Male: 21 (75)/20 (69) BMI: 18.5–29: 13 (46)/9 (31) ≥ 30: 15 (54)/20 (69) AHI: 35 (19)/41 (24) ESS: 7.9 (3)/7.3 (4)	12 months

Lai 2014	RCT	Ho Ting Shek Sleep Disorders Centre, Queen Mary Hospital, Hong Kong, China	OSA	49/51	CPAP	Age: 53 (10)/51 (10) Male: 41 (84)/42 (82) BMI: 28.6 (5.5)/29.3 (5.4) AHI: 30.7 (20.6, 52.2)/28.2 (20.3, 53.6) ESS: 9.5 (5.8)/9.0 (5.0)	3 months

Trupp 2011	RCT	Ohio State University Medical Center, Columbus, United States	OSA + CVD	36/34	CPAP	Age: 54.3 (29–74)/56.5 (38–77) Male: 19 (73)/17 (59) BMI: 32.9 (7.2)/35.0 (7.5) AHI: 37.5 (28.6)/42.4 (26.3) ESS: 9.4 (4.7)/10.3 (5.0)	30 days

Yu 2024	RCT	Respiratory clinics of two regional hospitals, Hong Kong, China	CHRF	62/62	NR	Age: 70.05 (11.20)/67.34 (14.23) Male: 27 (43.5)/30 (48.4) BMI: NR AHI: NR ESS: NR	12 months

Abbreviations: AHI, apnea–hypopnea index; BMI, body mass index; CG, control group; CHRF, chronic hypercapnic respiratory failure; CPAP, continuous positive airway pressure; CVD, cardiovascular disease; ESS, Epworth Sleepiness Scale; IG, intervention group; NIV, noninvasive ventilation; NR, not reported; OSA, obstructive sleep apnea; OSAS, obstructive sleep apnea syndrome; PAP, positive airway pressure; RCT, randomized controlled trial; SAHS, sleep apnea–hypopnea syndrome.

### 3.2. Nurse‐Led Interventions for NIV Adherence in Adult Patients in Included Studies

Across included studies, nurse‐led interventions for NIV adherence were generally multicomponent and could be grouped into three main domains: educational and informational support, motivational and behavioral strategies, and follow‐up, skills‐based, and telehealth interventions (see Table 2).

**TABLE 2:** Nurse‐led interventions in the included studies.

Study (author, year)	Intervention and comparator	Intervention content
Srikajon 2025	IG: short video‐based educational intervention CG: usual care	1. Basic information on OSA (1 min 20 s); 2. CPAP benefits (1 min); 3. Patient testimonials to enhance motivation (40 s); 4. Brief guidance on CPAP adverse effects and troubleshooting (30 s).

Aloia 2013	IG1: MET IG2: ED CG: usual care	IG1: 1. Assessing readiness and confidence to change and exploring the reasons for choosing that value; 2. Assessment of patient knowledge, permission‐based feedback, information provision, and elicitation of patient interpretation; 3. Perceived benefits of PAP in order to enhance outcome expectations; 4. Goal setting, consistent with both motivational interviewing style and social cognitive theory; 5. Identification of rewards for hard work on adherence to PAP; 6. Discussion of important values and goals and the ways in which PAP adherence facilitates and hinders these goals. 7. Two 45‐min face‐to‐face counseling sessions at Week 1 and Week 2 after PAP initiation; one booster phone call at Week 3. IG2: 1. Standardized education on apnea pathophysiology, medical and behavioral consequences, and treatment benefits, delivered using charts and diagrams (not tailored to readiness to change). 2. Two 45‐min face‐to‐face counseling sessions at Week 1 and Week 2 after PAP initiation; one booster phone call at Week 3.

Hoy 1999	IG: intensives support group CG: usual care	1. Home‐based initial CPAP education with involvement of the patient’s partner; 2. Additional CPAP titration in the sleep center for 2 extra nights (total 3 nights) to address early CPAP‐related problems; 3. Scheduled home visits by sleep nurses to the patient and partner at 7, 14, and 28 days and at 4 months after CPAP initiation to minimize treatment‐related problems.

Khan 2022	IG: I‐CPAP program CG: usual care	All intervention participants and their caregivers attended a group visit after PAP initiation, consisting of four structured patient‐centered sessions: 1. Interactive education on the health effects of OSA and benefits of PAP use; 2. Peer coaching by a patient with chronic OSA using PAP; 3. Hands‐on training with a respiratory therapist; 4. A semistructured 1‐on‐2 motivational interview.

Kooij 2021	IG: video consultation CG: usual care	1. A nurse‐led Face‐to‐talk video consultation intervention was initiated 1 week after CPAP initiation, targeting CPAP adherence, residual AHI, and symptom improvement. 2. Visit frequency was adaptively adjusted based on goal achievement (if targets were met, follow‐up occurred at 4 weeks; if not met, weekly video consultations were conducted until Week 4).

Rudilla 2021	IG: MEntA CG: usual care	A motivational interviewing–based CPAP school intervention for therapy initiation, delivered in a 90‐min session, consisting of two components: 1. Educational block: Expert explanation of sleep apnea and symptoms, peer demonstration of CPAP treatment and optimization, supported by printed and digital materials. 2. Training block: Hands‐on practice with treatment, equipment, safety, and hygiene, including mask fitting and equipment management. Educational and practical contents were delivered simultaneously.

Miller 2024	IG: MENDS CG: usual care	1. Intervention format: delivered virtually in four sessions (Weeks 2, 4, 6, and 8), each consisting of a 30–45‐min interactive discussion between the interventionist and the participant. 2. Session 1 (Week 2): conducted 1 week after CPAP initiation to target adherence‐related issues identified from device downloads using a motivational enhancement approach. 3. Data review and device support (each session): CPAP adherence data were reviewed, including average daily and weekly use, current AHI, average CPAP pressure, and mask leak. The interventionist provided device‐related support for mask, pressure, and equipment issues and coordinated pressure adjustments or new masks when needed. 4. Motivational intervention and tailoring: an individualized motivational intervention was delivered at each session, tailored to the participant’s activation level assessed by the Patient Activation Measure. The next session was scheduled after each visit with an email calendar invitation.

Sawyer 2019	IG: tailored intervention CG: usual care	1. SEMSA was assessed at baseline, before PAP titration polysomnography, on Day 2 of home PAP treatment, and between Days 7 and 10 of home PAP treatment to determine appropriate exposure content and timing. Daily symptom responses to PAP (daytime sleepiness and functional outcomes) were also assessed and used to address treatment outcome expectancies. 2. Each phase lasted 30–60 min and was delivered either at the sleep center or by telephone during home PAP treatment. Content addressed cognitive perceptions of diagnosis and treatment, outcome expectancies, and PAP self‐efficacy. Tailoring was guided by SEMSA subdomain scores (≤ 3.0); domains with scores > 3.0 were not targeted. When only risk perception met the threshold and outcome expectancy was ≤ 3.0, outcome expectancies related to participants’ comorbidities were addressed without emphasizing health risks of OSA. 3. Participant‐identified proximate social sources were consulted during the first treatment Week to provide observed treatment responses to participants. 4. Completed telephone visits on Days 2 and 5 of home PAP treatment and attended a 1‐week in‐person research visit with the interventionist.

Smith 2009	IG: habit‐promoting experimental audio intervention CG: placebo group	The CPAP intervention packet consisted of four components: 1. A 20‐min audio program providing instructions on comfortable mask fitting, correct hose connection, and relaxation despite positive airway pressure and noise; 2. Informational handouts describing the health consequences of nonuse and the benefits of adherence, plus an audio disc with relaxing music to facilitate sleep; 3. CPAP use reminder placards; 4. A 4‐week diary for recording CPAP use and documenting user experiences.

Chen 2015	IG: nurse‐led intensive intervention CG: usual care	1. Hospital health education: a 30‐min educational video explaining the pathogenesis, mechanisms, risks, benefits, and treatment of SAHS, along with individualized nurse guidance and distribution of a health education manual. 2. Patient self‐management interventions: nurses conducted home visits after CPAP initiation to reduce treatment‐related problems. Within 5 days of CPAP treatment, a 15‐min telephone interview was conducted to identify and resolve problems. Patients were advised on lifestyle modification. Psychological support focused on maintaining a positive mental state and teaching coping strategies for anxiety and depression. 3. Sleep physician consultation: within 1 month after CPAP initiation, patients attended a 30‐min consultation.

Parthasarathy 2025	IG: PDI‐IVR system CG: usual care	1. Trained peer buddies with OSA were matched to participants by age, sex, race/ethnicity, socioeconomic status, language (Spanish), and educational background; peers shared coping strategies based on their training and a peer buddy manual over 6 months. Peers also assisted with navigating the healthcare system and care coordination via IVR upon patient request. 2. Ten mandatory interactions were delivered in the first 3 months (2 in person and 8 by telephone), each ≤ 30 min. In the subsequent 3 months, up to eight optional, on‐demand peer interactions were available, each ≤ 30 min. 3. Interactions included two supervised in‐person sessions (Days 1 and 7) and telephone contacts via an IVR system (weekly for 1 month, then fortnightly).

Wang 2012	IG1: education IG2: PMR IG3: education and PMR CG: usual care	IG1: 1. Attended a 4‐h group education session on OSA and CPAP during the first week. 2. Underwent three nights of in‐hospital CPAP titration to address treatment‐related problems. 3. Received educational materials, including a brochure on the need for and benefits of CPAP therapy and a 20‐min instructional video on CPAP device use. 4. Had access to a 24‐h telephone consultation service with sleep nurses. IG2: 1. Received one night of in‐hospital CPAP titration at study entry. 2. Participated in PMR training for 12 weeks (one 40‐min group session per week). 3. Received educational materials (a brochure on relaxation mechanisms and benefits and an audio CD for at‐home PMR practice) and were instructed to practice PMR at home before CPAP use and record relaxation experiences. 4. At‐home PMR practice was reviewed at the beginning of each weekly session with discussion of problems with the sleep nurse. IG3: 1. Received three nights of in‐hospital CPAP titration at study entry and both educational and PMR interventions. 2. During the first study week, the first PMR training session and the 4‐h group education session were delivered on separate days.

Martinotti 2025	IG: telemonitoring CG: usual care	1. Followed via telemonitoring using data downloads through the ResMed Air‐View platform. 2. Received telephone contacts every 30 days for 180 days and every 60 days thereafter up to 365 days.

Lai 2014	IG: brief motivational enhancement education program CG: usual care	1. Viewed a 25‐min video featuring the real‐life experience of a CPAP user and received an information booklet on OSA and CPAP. 2. Received a 20‐min patient‐centered face‐to‐face brief motivational interview the morning after CPAP titration, using importance and confidence rulers to explore barriers and facilitators of CPAP use, applying a decision matrix to discuss the pros and cons of CPAP use versus nonuse, and addressing expected outcomes and benefits of CPAP therapy. 3. Received a 10‐min telephone call from the same interviewer on Day 2 of CPAP use.

Trupp 2011	IG: positive educational message intervention CG: negative educational message intervention	1. Viewed a brief (50‐s) educational video on CPAP and its benefits. 2. Received a magnet reinforcing the assigned educational message and were instructed to place it in a prominent location for frequent reference. 3. Received brief weekly telephone calls (1–2 min) for 4 weeks reinforcing the same educational message.

Yu 2024	IG: IMB–NIV program CG: usual care	1. Initial home visit (60 min): Nurse assessed self‐care needs related to CHRF and NIV management; provided health education to increase awareness of nonadherence, misbeliefs, and adverse health consequences to support goal setting and action planning; applied a nurse–patient partnership approach to address concerns, challenge negative thoughts, and identify personal and social support resources; delivered skills training on proper NIV use and complication prevention in the home setting; discussed potential barriers and solutions; involved family members when available; and offered an office‐hours telephone consultation hotline. 2. Tele‐visits (Weeks 2 and 4, 20 min each): Monitored progress and reviewed goals; provided health counseling to address barriers to NIV adherence and emerging self‐care challenges. 3. Follow‐up clinic visit (30 min): Reinforced long‐term behavioral change; encouraged reflection on improvements in NIV self‐care and perceived health benefits; and provided additional skills training, positive reinforcement of prognostic benefits, and practical strategies for integrating self‐care into daily routines.

Abbreviations: CD, compact disc; CG, control group; CHRF, chronic hypercapnic respiratory failure; CPAP, continuous positive airway pressure; ED, education; h, hour(s); I‐CPAP program, improving CPAP adherence program; IG, intervention group; IMB–NIV program, information–motivation–behavioral skills–based noninvasive ventilation program; IVR, interactive voice response; MENDS, motivational enhancement and device support; MEntA, motivational interviewing–based intervention; MET, motivational enhancement therapy; NIV, noninvasive ventilation; OSA, obstructive sleep apnea; PAP, positive airway pressure; PDI–IVR system, peer‐driven intervention via an interactive voice response system; PMR, progressive muscle relaxation; SAHS, sleep apnea–hypopnea syndrome; SEMSA, Self‐efficacy Measure in Sleep Apnea.

#### 3.2.1. Educational and Informational Interventions

Most nurse‐led interventions incorporated educational components designed to enhance patients’ understanding of OSA or CHRF, NIV therapy, and the benefits of adherence. These interventions typically delivered basic information on disease mechanisms, treatment rationale, and the potential health consequences of nonuse through videos, printed materials, or face‐to‐face instruction. For example, brief educational videos and written materials were used to convey key messages regarding CPAP use and its benefits [21–25]. Several studies supplemented educational content with demonstrations of device operation and hands‐on training in mask fitting and equipment management [26–29]. Educational sessions were delivered individually or in group formats and were often initiated shortly after NIV or CPAP commencement, emphasizing early knowledge acquisition to support subsequent adherence [26, 30, 31].

#### 3.2.2. Motivational and Behavioral Interventions

Beyond information provision, many nurse‐led interventions explicitly targeted motivational and cognitive determinants of adherence using patient‐centered and theory‐informed approaches. Motivational interviewing techniques were frequently employed to explore readiness for change, clarify personal values and goals, and address perceived barriers to NIV use [24–27]. Tailored interventions grounded in self‐efficacy, outcome expectancy, and illness perceptions were also implemented, with intervention content adapted according to validated instruments such as the Self‐efficacy Measure in Sleep Apnea (SEMSA) or the Patient Activation Measure [32, 33]. These programs commonly incorporated goal setting, reinforcement of perceived benefits, and individualized feedback derived from device‐generated data. Peer‐based motivational strategies were integrated in some programs, whereby trained patients with OSA provided experiential support and role modeling to enhance engagement and treatment persistence [21, 27, 34].

#### 3.2.3. Follow‐Up, Skills Support, and Telehealth Interventions

Ongoing follow‐up and practical problem‐solving constituted another core component of nurse‐led adherence interventions. Nurses provided continued support through home visits, telephone calls, or video consultations to identify and resolve device‐related problems, including mask discomfort, air leakage, and persistent symptoms [23, 29, 30, 35]. Several studies incorporated telemonitoring platforms to review adherence data and respiratory parameters and to guide timely adjustment of treatment strategies or counseling intensity [33, 36]. Family members or caregivers were actively involved in selected interventions to reinforce daily self‐care behaviors and to address psychosocial barriers to NIV use [29, 30, 32]. These follow‐up strategies were generally initiated during the early treatment phase and extended over weeks to months.

### 3.3. Effects of Nurse‐Led Interventions on Adherence to NIV in Adult Patients

Across the included studies, nurse‐led interventions demonstrated heterogeneous effects on NIV adherence across short‐term (≤ 1 month), medium‐term (1–6 months), and long‐term (6–12 months) follow‐up periods (see Table 3).

**TABLE 3:** Adherence and other clinical outcomes reported in the included studies.

Study (author, year)	Role of adherence	Other clinical outcomes	Adherence‐related results (IG vs. CG)
Srikajon 2025	Primary outcome	NR	3 weeks: CPAP use ≥ 4 h/night on ≥ 70% of nights: 68.2% vs. 50%, ***p* = 0.034**; Mean nightly CPAP use: 6.0 vs. 4.5 h, ***p* = 0.012**; Percent of days with CPAP use ≥ 4 h: 87.4% vs. 68.9%, ***p* = 0.005**. 3 months: Adherent: 55.1% vs. 14.8%; Nonadherent: 20.5% vs. 22.2%; Loss to follow‐up: 24.4% vs. 63%.

Aloia 2013	Primary outcome	Decisional balance, self‐efficacy	Mean nightly PAP use: 1 month: (IG1 *n* = 62, IG2 *n* = 67, CG *n* = 54): 4.26 ± 2.27 vs. 4.43 ± 2.51 vs. 3.82 ± 2.27 h; 2 months (IG1 *n* = 62, IG2 *n* = 67, CG *n* = 55): 4.37 ± 2.34 vs. 4.48 ± 2.48 vs. 3.68 ± 2.37 h; 3 months (IG1 *n* = 62, IG2 *n* = 66, CG *n* = 55): 4.34 ± 2.35 vs. 4.48 ± 2.50 vs. 3.65 ± 2.36 h; 6 months (IG1 *n* = 58, IG2 *n* = 56, CG *n* = 55): 4.14 ± 2.34 vs. 4.34 ± 2.41 vs. 3.65 ± 2.46 h; 12 months (IG1 *n* = 47, IG2 *n* = 53, CG *n* = 49): 3.86 ± 2.61) vs. 4.34 ± 2.45 vs. 3.73 ± 2.50 h.

Hoy 1999	Primary outcome	Severity of sleep apnea, symptoms, mood, cognitive function, sleepiness, QoL	Mean nightly CPAP use: 6 months: 5.4 ± 0.3 vs. 3.8 ± 0.4 h, ***p* = 0.003**.

Khan 2022	Primary outcome	ESS; CPAP tactics survey, pre‐ and postassessment surveys, COMRADE survey	PAP use ≥ 4 h/day on ≥ 70% of days: Baseline: 46% (95% CI: 28–65) vs. 47% (95% CI: 30–64); 3 months: 50% (95% CI: 32–68) vs. 43% (95% CI: 27–61); 6 months: 54% (95% CI: 35–72) vs. 40% (95% CI: 24–58); *p* (time × group interaction) = 0.59 (unadjusted), 0.46 (age‐adjusted). Mean daily PAP use: Baseline: 4.2 h (95% CI: 3.3–5.2) vs. 4.9 h (95% CI: 4.0–5.9); 3 months: 4.1 h (95% CI: 3.1–5.2) vs. 4.6 h (95% CI: 3.7–5.5); 6 months: 4.6 h (95% CI: 3.6–5.7) vs. 4.0 h (95% CI: 2.9–5.0); *p* (time × group interaction) = 0.43 (unadjusted), **0.03** (age‐adjusted).

Kooij 2021	Primary outcome	SEMSA; relation between self‐efficacy, risk perception, outcome expectancies, and CPAP use; expectations and experiences with video consultation; satisfaction	Mean nightly CPAP use: 4 weeks: 334.4 min (95% CI: 302.1–366.5) vs. 371.4 min (95% CI: 340.1–402.7); 12 weeks: 311.5 min (95% CI: 278.4–344.6) vs. 348.6 min (95% CI: 316.5–380.7); 24 weeks: 295.2 min (95% CI: 260.0–330.4) vs. 332.7 min (95% CI: 298.1–366.5).

Rudilla 2021	Primary outcome	Motivation, perceived competence, QoL, sleepiness, emotional state, daily activities, and social relationships	Mean nightly CPAP use: 30 days (mean difference): 0.48 h (95% CI: −0.69–1.67), *p* = 0.413; 90 days (mean difference): 1.60 h (95% CI: 0.60–2.61), ***p* < 0.01**; 90 days (RR): 4.57 h (95% CI: 1.67–12.51), ***p* < 0.001**.

Miller 2024	Secondary outcome	Nine feasibility outcomes: daytime sleepiness, physical, mental, and social health, cognition, AHI, self‐efficacy, and patient activation	Percent of nights with CPAP use ≥ 4 h: 1 week: 72% vs. 77%; 2 weeks: 77% vs. 82%; 3 weeks: 81% vs. 86%; 4 weeks: 82% vs. 72%; 5 weeks: 82% vs. 71%; 6 weeks: 84% vs. 72%; 7 weeks: 77% vs. 71%; 8 weeks: 78% vs. 67%; 9 weeks: 82% vs. 69%; 10 weeks: 75% vs. 71%; 11 weeks: 76% vs. 68%; 12 weeks: 71% vs. 68%. Mean weekly CPAP use: 1 week: 38.8 vs. 44.0 h; 2 weeks: 39.7 vs. 41.9 h; 3 weeks: 40.6 vs. 44.3 h; 4 weeks: 40.4 vs. 39.0 h; 5 weeks: 41.8 vs. 39.0 h; 6 weeks: 42.7 vs. 36.4 h; 7 weeks: 39.9 vs. 36.1 h; 8 weeks: 40.4 vs. 35.8 h; 9 weeks: 39.8 vs. 35.6 h; 10 weeks: 37.6 vs. 39.1 h; 11 weeks: 38.1 vs. 37.9 h; 12 weeks: 34.8 vs. 36.3 h. Mean daily CPAP use: 1 week: 5.83 vs. 6.86 h; 2 weeks: 6.16 vs. 6.39 h; 3 weeks: 6.25 vs. 6.55 h; 4 weeks: 6.35 vs. 6.24 h; 5 weeks: 6.20 vs. 5.96 h; 6 weeks: 6.42 vs. 5.87 h; 7 weeks: 5.95 vs. 5.56 h; 8 weeks: 6.06 vs. 5.38 h; 9 weeks: 5.92 vs. 5.56 h; 10 weeks: 5.92 vs. 5.92 h; 11 weeks: 5.96 vs. 5.89 h; 12 weeks: 5.54 vs. 5.73 h.

Sawyer 2019	Primary outcome	Self‐efficacy	Mean nightly PAP use: 7 days: 6.4 ± 2.01 vs. 5.8 ± 1.35 h, *p* = 0.20; 30 days: 5.1 ± 2.05 vs. 5.1 ± 1.66 h, *p* = 0.90; 90 days: 4.8 ± 2.27 vs. 4.7 ± 1.85 h, *p* = 0.89.

Smith 2009	Primary outcome	Written diary data, patient satisfaction survey	CPAP use ≥ 4 h/night at least 9 of each 14 nights: 1 month: 89.0% vs. 54.9%, *X* ^2^ = 14.67, ***p* < 0.01**; 3 months: 54.5% vs. 57.1%, *X* ^2^ = 0.065, *p* = 0 0.79; 6 months: 74.5% vs. 71.4%, *X* ^2^ = 0.118, *p* = 0.73.

Chen 2015	Primary outcome	Symptoms, daytime sleepiness, mood, QoL	Mean nightly CPAP use: 1 month: 6.32 ± 1.25 vs. 4.35 ± 1.71 h, ***p* < 0.05**; 3 months: 6.41 ± 1.30 vs. 4.20 ± 1.20 h, ***p* < 0.01**; 6 months: 6.31 ± 1.81 vs. 4.31 ± 1.25 h, ***p* < 0.01**; 12 months: 6.67 ± 1.36 vs. 4.02 ± 1.23, ***p* < 0.01**.

Parthasarathy 2025	Primary outcome	Satisfaction; patient assessment of care for chronic conditions; client perception of care coordination; patient’s satisfaction with care; patient knowledge, skill, and confidence for self‐management; patient perceptions related to treatment adherence; HR–QoL	6 months: Mean nightly CPAP use: 4.6 ± 0.1 vs. 3.8 ± 0.1 h, ***p* < 0.0001** (unadjusted ITT); 4.5 ± 0.2 vs. 3.7 ± 0.2 h, ***p* < 0.01** (age, sex, nasal congestion score‐adjusted ITT). Percent of nights with CPAP use > 4 h: 62.3 ± 1.7 vs. 52.0 ± 1.8, ***p* < 0.0001** (unadjusted ITT); 62.0 ± 3.0 vs. 51.4 ± 3.0, **p < 0.05** (age, sex, nasal congestion score‐adjusted ITT). CPAP use > 4 h/night on > 70% of nights: 52.1% vs. 39.9%, ***p* ≤ 0.001** (unadjusted ITT); OR = 1.6 (95% CI: 1.2–2.2), **p < 0.01** (age, sex, nasal congestion score‐adjusted ITT).

Wang 2012	Primary outcome	Daytime sleepiness, sleep quality, anxiety, depression	CPAP use ≥ 4 h/night at least 9 of each 14 nights: 4 weeks (IG1 vs. IG2 vs. IG3 vs. CG): 89.5% vs. 86.8% vs. 92.1% vs. 60.5%; 8 weeks (IG1 vs. IG2 vs. IG3 vs. CG): 71.1% vs. 76.3% vs. 86.8% vs. 55.3%; 12 weeks (IG1 vs. IG2 vs. IG3 vs. CG): 60.5% vs. 68.4% vs. 78.9% vs. 47.4%. Paired comparison of CPAP use ≥ 4 h/night at least 9 of each 14 nights: 4 weeks (IG1 vs. CG): *χ* ^2^ = 8.491, ***p* = 0.004**; 4 weeks (IG2 vs. CG): *χ* ^2^ = 6.786, ***p* = 0.009**; 4 weeks (IG3 vs. CG): *χ* ^2^ = 10.483, ***p* = 0.001**; 8 weeks (IG1 vs. CG): *χ* ^2^ = 2.036, *p* = 0.154; 8 weeks (IG2 vs. CG): *χ* ^2^ = 3.7415, *p* = 0.053; 8 weeks (IG3 vs. CG): *χ* ^2^ = 9.212, ***p* = 0.002**; 12 weeks (IG1 vs. CG): *χ* ^2^ = 1.324, *p* = 0.250; 12 weeks (IG2 vs. CG): *χ* ^2^ = 3.455, *p* = 0.063; 12 weeks (IG3 vs. CG): *χ* ^2^ = 8.143, ***p* = 0.004**.

Martinotti 2025	Primary outcome	Appropriate CPAP use and the number of in‐person healthcare services provided; daytime sleepiness; treatment effectiveness based on residual AHI	Percent of days with CPAP use: 180 days: 89% vs. 92%; 365 days: 92% vs. 93%. Mean nightly CPAP use: 180 days: 5.30 ± 1.6 vs. 5.56 ± 1.6 h; 365 days: 5.65 ± 1.2 vs. 5.53 ± 1.9 h.

Lai 2014	Primary outcome	Adherence‐related cognitions, daytime sleepiness, HR–QoL	Mean daily CPAP use: 1 week: 5.5 ± 1.8 vs. 2.9 ± 2.5 h; 1 month: 4.8 ± 1.6 vs. 2.6 ± 2.3 h; 3 months: 4.4 ± 1.8 vs. 2.4 ± 2.3 h; Between‐group difference (estimate): 2.0 (95% CI: 1.3–2.8), ***p* < 0.001**. CPAP use ≥ 4 h/day on ≥ 70% of days: 1 week: 63% vs. 29%; 1 month: 59% vs. 20%; 3 months: 41% vs. 20%; Between‐group difference (OR): 4.3 (95% CI: 2.0–9.0), ***p* < 0.001**. Percent of days with CPAP use ≥ 4 h: 1 week: 74 ± 29 vs. 39 ± 38; 1 month: 70 ± 26 vs. 34 ± 33; 3 months: 61 ± 28 vs. 32 ± 32; Between‐group difference (estimate): 31 (95% CI: 19–43), ***p* < 0.001**.

Trupp 2011	Primary outcome	Daytime sleepiness, dispositional optimism, depression, self‐efficacy	30 days: Percent of nights with CPAP use ≥ 4 h: 42.15% vs. 63.14%; CPAP use ≥ 4 h/night on ≥ 70% of nights: 23% vs. 55%, *χ* ^2^ (1, *n* = 55) = 5.88, ***p* = 0.015**.

Yu 2024	Primary outcome	Sleep quality, HR–QoL; venous bicarbonate (HCO_3_^−^) level, emergency department admissions, hospital admissions	Percent of days with NIV use > 4 h: Baseline: 41.32 ± 33.17 vs. 44.05 ± 33.93; 7 weeks: 68.90 ± 35.49 vs. 43.31 ± 38.80; Time × group interaction effect by GEE: *B* = 27.70 (95% CI: 16.09–39.30), ***p* < 0.001**. 3 months: 67.22 ± 37.98 vs. 43.08 ± 41.69; Time × group interaction effect by GEE: *B* = 27.23 (95% CI: 14.30–40.16), ***p* < 0.001**. 6 months: 65.93 ± 40.27 vs. 43.79 ± 40.00; Time × group interaction effect by GEE: *B* = 26.86 (95% CI: 13.87–39.85), ***p* < 0.001**. 12 months: 72.46 ± 35.14 vs. 45.05 ± 37.51; Time × group interaction effect by GEE: *B* = 29.37 (95% CI: 17.33–41.40), ***p* < 0.001**. Mean daily NIV use: Baseline: 2.95 ± 1.83 vs. 2.87 ± 1.83 h. 7 weeks: 5.16 ± 2.59 vs. 2.96 ± 1.83 h; Time × group interaction effect by GEE: *B* = 2.09 (95% CI: 1.38–2.81), ***p* < 0.001**. 3 months: 5.13 ± 2.93 vs. 3.16 ± 2.92 h; Time × group interaction effect by GEE: *B* = 1.94 (95% CI: 1.08–2.80), ***p* < 0.001**. 6 months: 5.19 ± 2.99 vs. 3.24 ± 3.08 h; Time × group interaction effect by GEE: *B* = 2.03 (95% CI: 1.05–3.01), ***p* < 0.001**. 12 months: 5.69 ± 3.03 vs. 3.24 ± 2.83 h; Time × group interaction effect by GEE: *B* = 2.37 (95% CI: 1.44–3.31), ***p* < 0.001**. NIV use > 4 h/d on > 70% of days: 7 weeks: 51.6% vs. 16.1%; Time × group interaction effect by GEE: *B* = 3.13 (95% CI: 1.69–5.88), ***p* < 0.001**. 3 months: 56.5% vs. 29.0%; Time × group interaction effect by GEE: *B* = 1.88 (95% CI: 1.20–2.94), ***p* = 0.003**. 6 months: 58.1% vs. 22.6%; Time × group interaction effect by GEE: *B* = 2.50 (95% CI: 1.52–4.17), ***p* < 0.001**. 12 months: 61.3% vs. 27.4%; Time × group interaction effect by GEE: *B* = 2.78 (95% CI: 1.69–4.55), ***p* < 0.001**.

*Note:* The bold values indicate statistically significant results (*p* < 0.05).

Abbreviations: *χ* ^2^, chi‐square; 95% CI, 95% confidence interval; AHI, apnea–hypopnea index; *B*, regression coefficient; CD, compact disc; CG, control group; COMRADE, Combined Outcome Measure for Risk Communication and Treatment Decision‐Making Effectiveness; CPAP, continuous positive airway pressure; ESS, Epworth Sleepiness Scale; GEE, generalized estimating equation; h, hour(s); HR–QoL, health‐related quality of life; I‐CPAP program, improving CPAP adherence program; IG, intervention group; IMB program, information–motivation–behavioral; ITT, intention‐to‐treat; MENDS, motivational enhancement and device support; MEntA, motivational interviewing–based intervention; MET, motivational enhancement therapy; NIV, noninvasive ventilation; NR, not reported; OR, odds ratio; *p*, *p* value; PAP, positive airway pressure; PDI–IVR system, peer‐driven intervention via an interactive voice response system; PMR, progressive muscle relaxation; RR, relative risk; SEMSA, Self‐efficacy Measure in Sleep Apnea.

In the short term, several nurse‐led interventions demonstrated significant improvements in NIV adherence compared with usual care, although null findings were also reported. A brief video‐based educational intervention significantly increased the proportion of patients achieving CPAP use ≥ 4 h/night on ≥ 70% of nights at 3 weeks (68.2% vs. 50%, *p* = 0.034), as well as mean nightly use (6.0 vs. 4.5 h, *p* = 0.012) [21]. Similarly, a brief motivational enhancement program delivered by nurses resulted in higher mean daily CPAP use at 1 week (5.5 ± 1.8 vs. 2.9 ± 2.5 h) and 1 month (4.8 ± 1.6 vs. 2.6 ± 2.3 h), with a significant between‐group difference (*p* < 0.001) [25]. Nurse‐led intensive education combined with early follow‐up also significantly increased mean nightly CPAP use at 1 month (6.32 ± 1.25 vs. 4.35 ± 1.71 h, *p* < 0.05) [23]. In contrast, a nurse‐led teleconsultation intervention showed no improvement in mean nightly CPAP use at 4 weeks (334.4 vs. 371.4 min) [35], and a tailored cognitive intervention did not significantly improve mean PAP use at 7 and 30 days [32]. Moreover, a positive educational message intervention paradoxically resulted in lower short‐term adherence compared with a negative message (CPAP use ≥ 4 h/night on ≥ 70% of nights: 23% vs. 55%, *p* = 0.015) [22].

At medium‐term follow‐up, nurse‐led interventions produced mixed effects on adherence outcomes. Several multicomponent or motivationally oriented programs achieved statistically significant improvements. A motivational interviewing–based CPAP school intervention significantly increased mean nightly CPAP use at 3 months (mean difference 1.60 h, 95% CI 0.60–2.61, *p* < 0.01) [26]. Nurse‐led intensive intervention significantly improved mean nightly CPAP use at both 3 months (6.41 ± 1.30 vs. 4.20 ± 1.20 h, *p* < 0.01) and 6 months (6.31 ± 1.81 vs. 4.31 ± 1.25 h, *p* < 0.01) [23]. A peer‐supported, nurse‐coordinated intervention significantly increased mean nightly CPAP use at 6 months (4.6 ± 0.1 vs. 3.8 ± 0.1 h, *p* < 0.0001) and the proportion of nights with use > 4 h (62.3% vs. 52.0%, *p* < 0.0001) [34]. In patients with chronic respiratory failure, an information–motivation–behavioral skills–based program (IMB–NIV program) significantly increased the percentage of days with NIV use > 4 h at 3 months (67.22% vs. 43.08%; *p* < 0.001) and 6 months (65.93% vs. 43.79%; *p* < 0.001) [29]. Conversely, some interventions did not yield significant between‐group differences. A group‐based patient‐centered intervention incorporating education and peer coaching failed to improve PAP adherence at three or 6 months (time × group interaction *p* = 0.59 and 0.46, respectively) [27]. Nurse‐led video consultation consistently resulted in lower mean nightly CPAP use in the intervention group than in controls at 12 and 24 weeks [35]. A tailored intervention similarly failed to demonstrate significant differences in mean PAP use at 90 days [32]. Educational and relaxation‐based interventions improved adherence at 4 weeks but showed diminishing effects at 8 and 12 weeks, with only the combined education and PMR group remaining significantly different from controls at 12 weeks (*p* = 0.004) [28].

Evidence for long‐term effects suggested that sustained nurse‐led interventions could maintain or further enhance adherence, although effect sizes were heterogeneous. In a randomized trial of an intensive nurse‐led intervention, mean nightly CPAP use remained significantly higher in the intervention group at 12 months (6.67 ± 1.36 vs. 4.02 ± 1.23 h, *p* < 0.01) [23]. Similarly, the IMB‐based nurse‐led program for chronic respiratory failure demonstrated sustained benefits at 12 months, with a higher percentage of days with NIV use > 4 h (72.46% vs. 45.05%; *p* < 0.001) and greater mean daily use (5.69 ± 3.03 vs. 3.24 ± 2.83 h; *p* < 0.001) [29]. By contrast, telemonitoring combined with periodic nurse contact did not result in significant differences in the percentage of days with CPAP use or mean nightly use at 180 or 365 days [36]. A motivational education trial also showed convergence between groups by 6 months, with no significant differences in the proportion of patients achieving CPAP use ≥ 4 h/night [31]. Long‐term follow‐up in a motivational counseling study demonstrated modest differences in mean nightly PAP use at 12 months, without clear superiority over standard education [24].

### 3.4. Effects of Nurse‐Led Interventions on Clinical Outcomes in Adult Patients Receiving NIV

Several studies examined the effects of nurse‐led interventions on symptom‐related outcomes, including daytime sleepiness, sleep quality, and QoL. Hoy et al. [30] reported significantly lower symptom scores at 6 months in the intensive support group compared with standard care (*p* = 0.03). Similarly, Chen et al. [23] observed significantly greater reductions in symptom scores at both 6 and 12 months in the nurse‐led intensive intervention group than in the usual care group (*p* < 0.01). Regarding daytime sleepiness, Wang et al. [28] demonstrated significant improvements in Epworth Sleepiness Scale (ESS) scores in the combined education and progressive muscle relaxation group compared with usual care at 4, 8, and 12 weeks, whereas education alone produced only short‐term effects. Yu et al. [29] further reported sustained improvements in sleep quality (Cohen’s *d* range, 0.59–0.92) and health‐related QoL (Cohen’s *d* range, 0.53–0.93) from 3 to 12 months in the IMB–NIV group. However, several studies did not detect statistically significant between‐group differences. Khan et al. [27] observed improvements in ESS scores in both the CPAP adherence improvement program and usual care groups, without preferential effects of the intervention. Parthasarathy et al. [34] likewise found no significant differences in ESS or disease‐specific QoL between groups after adjustment for confounders, although per‐protocol analyses suggested a small improvement in ESS that did not reach the minimal clinically important difference.

Psychological and cognitive‐behavioral outcomes were also frequently assessed. Nurse‐led interventions demonstrated beneficial effects on mood and emotional well‐being in several trials. Hoy et al. [30] reported significantly lower depression scores in the intensive support group than in the standard care group, whereas Chen et al. [23] found significantly greater improvements in mood in the intervention group over 12 months (*p* < 0.001). Rudilla et al. [26] observed significant improvements in emotional state and social relationships at 30 and 90 days in the motivational intervention group compared with usual care. With respect to self‐efficacy and decision‐related constructs, mixed findings were reported. Lai et al. [25] demonstrated a significant improvement in treatment self‐efficacy at 3 months in the motivational enhancement education group compared with controls (mean difference 0.2 units; *p* = 0.012), and Parthasarathy et al. [34] reported greater gains in self‐efficacy in the intervention arm (*p* < 0.001). In contrast, Kooij et al. [35] found no significant between‐group differences in self‐efficacy, outcome expectancies, or risk perception. Decision‐making outcomes improved in selected domains: Khan et al. [27] showed that several COMRADE items related to knowledge of treatment options and patient involvement were significantly associated with the intervention (odds ratios 3.25–3.65), whereas Aloia et al. [24] reported largely nonsignificant effects on decisional balance and self‐efficacy measures.

Health service utilization and patient satisfaction were reported in a limited number of studies. Yu et al. [29] found that the IMB–NIV program significantly reduced unplanned ED visits over 12 months (IRR 0.47; *p* = 0.01) and delayed time to respiratory‐related ED visits, although no significant differences were observed in hospital admissions. Subgroup analyses indicated that reductions in ED visits and hospital admissions were confined to patients with COPD. Parthasarathy et al. [34] reported significantly higher global satisfaction with CPAP support in the intervention group than in controls (*p* < 0.001) and more favorable patient assessments of chronic care (*p* = 0.009), with no significant differences in composite healthcare utilization. Other clinical and feasibility‐related outcomes showed variable results. Martinotti et al. [36] observed no significant differences between telemonitoring and usual care in mask fit or leak rates, although differences in the number of follow‐up visits were noted. Miller et al. [33] demonstrated the feasibility of delivering a nurse‐led telehealth intervention with minimal attrition but found no statistically significant between‐group differences in daytime sleepiness or cognitive outcomes.

### 3.5. Quality Appraisal Results

The risk‐of‐bias assessment indicated that 25% of the studies were rated as low risk, 56.3% as having some concerns, and 18.7% as high risk. All studies were judged to be at low risk in the domains of deviations from intended interventions and measurement of outcomes. Some studies were rated as having some concerns in the domain of selection of the reported results, primarily because study protocols were not preregistered. A small number of studies were rated as having some concerns in the randomization process domain, mainly due to the absence of blinding procedures. In addition, a few studies were rated as high risk in the missing outcome data domain, largely because of loss to follow‐up among participants (see Figure 2).

**FIGURE 2:**
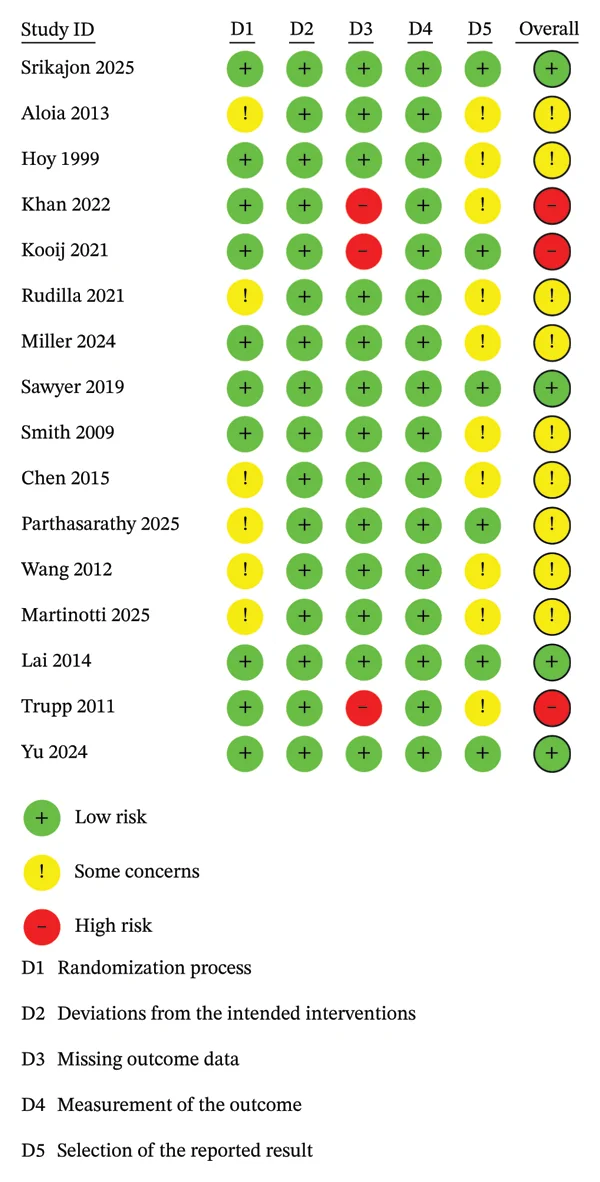
A summary of the risk of bias assessment for the included studies.

## 4. Discussion

This systematic review synthesizes evidence from 16 RCTs evaluating nurse‐led interventions to improve adherence and clinical outcomes among adults receiving NIV. Overall, nurse‐led interventions demonstrated a positive but heterogeneous effect on NIV adherence, with more consistent benefits observed in the short and medium term and sustained effects mainly achieved by intensive, theory‐informed, or multicomponent programs. Interventions combining educational components with motivational or behavioral strategies and structured follow‐up were generally more effective than information provision alone. Beyond adherence, nurse‐led interventions were associated with improvements in symptom burden, emotional well‐being, and selected health service outcomes, whereas effects on QoL, self‐efficacy, and healthcare utilization were inconsistent.

Our findings are broadly consistent with previous systematic reviews indicating that supportive and behavioral strategies are more effective than education alone in improving CPAP adherence. The Cochrane review by Askland et al. [14] reported that behavioral interventions produced clinically meaningful increases in nightly CPAP use, whereas supportive interventions yielded modest benefits and educational interventions were supported by very low‐certainty evidence. Similarly, López‐López et al. [12] found that educational and multidimensional nursing interventions improved CPAP adherence, underscoring the central role of nurses in supporting device use among patients with OSA. The observed effectiveness of motivational and psychologically informed nurse‐led interventions in our review is also consistent with meta‐analytic evidence demonstrating that motivational interviewing increases PAP adherence among treatment‐naive patients [15]. Moreover, our findings that telehealth‐ or eHealth‐supported interventions achieved short‐term improvements in adherence align with previous evidence that eHealth strategies increase average nightly CPAP use by approximately 0.5 h in the early months of treatment [11] and that virtual consultations are comparable to in‐person care in improving sleepiness and adherence [10].

In contrast to prior reviews suggesting consistent benefits of telemonitoring and virtual follow‐up [10, 11], several teleconsultation or telemonitoring interventions in our review did not demonstrate sustained improvements in adherence. This discrepancy may reflect differences in intervention intensity, degree of personalization, and integration of behavioral components. While technology‐enabled care can facilitate early problem detection and improve convenience, its effectiveness appears to depend on the provision of active behavioral support rather than passive data transmission alone. Furthermore, although psychosocial and behavioral interventions have been shown to improve sleep quality and emotional outcomes [9], effects on QoL and self‐efficacy were inconsistent in our review, echoing the uncertainty reported in the Cochrane analysis [14]. These mixed findings may be attributable to variability in outcome measurement, short follow‐up durations, and limited statistical power for secondary endpoints.

The observed benefits of nurse‐led interventions may be explained by their capacity to address psychological and contextual determinants of NIV adherence. According to the biopsychosocial and the predisposing, precipitating, and perpetuating factors model (3P model), adherence is shaped by predisposing factors (e.g., health literacy and beliefs), precipitating factors (e.g., device‐related anxiety and stigma), and perpetuating factors (e.g., ongoing discomfort and suboptimal patient–provider communication) [8]. Interventions incorporating motivational interviewing, tailored feedback, and skills training directly target these mechanisms by enhancing perceived benefits, self‐efficacy, and coping strategies [9, 15]. Nurse‐led follow‐up further enables early troubleshooting and emotional reassurance, thereby reducing barriers related to mask discomfort, uncertainty, and treatment burden, which are key contributors to early discontinuation.

The heterogeneity in intervention effects observed across trials may partly reflect methodological variability in intervention design, theoretical grounding, and outcome assessment. Evidence suggests that adherence interventions grounded in behavior change theory are more likely to be effective [13]; however, only a subset of the included trials explicitly adopted theoretical frameworks such as motivational interviewing or the 3P model. In addition, differences in follow‐up duration, intensity of nurse contact, and mode of delivery (face‐to‐face vs. telehealth) likely contributed to divergent results. Risk‐of‐bias concerns related to randomization procedures and incomplete outcome data further limit confidence in the overall conclusions, consistent with findings from previous adherence intervention reviews [14].

These findings highlight the pivotal role of nurses in delivering effective adherence support for adults receiving NIV. Interventions that integrate education with motivational and behavioral strategies and provide structured follow‐up should be prioritized in clinical practice. Nurse‐led models are particularly well suited to the early phase of NIV initiation, when adherence patterns are established and patients are most vulnerable to discontinuation. The incorporation of telehealth and eHealth tools may extend the reach of nurse‐led care, especially in resource‐limited or geographically remote settings, but should be embedded within patient‐centered and theory‐informed frameworks rather than implemented as stand‐alone solutions [10, 11]. Tailoring interventions to patients’ psychological profiles and disease contexts may further enhance effectiveness and optimize healthcare utilization.

## 5. Conclusion

This systematic review suggests that nurse‐led interventions may improve adherence to NIV among adult patients, particularly when education is combined with motivational and behavioral strategies and structured follow‐up. However, evidence regarding the long‐term effects on NIV adherence remains limited, and the effects on clinical outcomes remain inconsistent.

## 6. Limitations

This review has several limitations. First, substantial heterogeneity in intervention content, intensity, and outcome definitions precluded quantitative synthesis. Second, most included studies focused on populations with OSA, limiting generalizability to patients receiving NIV for other indications, such as chronic respiratory failure. Third, risk‐of‐bias assessments indicated that a considerable proportion of studies had methodological concerns, particularly related to incomplete outcome data and selective reporting. Fourth, long‐term outcomes beyond 12 months were rarely reported, and evidence regarding sustained effects remains limited. Finally, publication bias cannot be excluded, as studies reporting null or negative findings may be underrepresented.

## Funding

No funding was received for this research.

## Conflicts of Interest

The authors declare no conflicts of interest.

## Supporting Information

Additional supporting information can be found online in the Supporting Information section.

## Supporting information


**Supporting Information 1** Supporting File 1 provides the detailed search strategies for all databases used in this study.

**Supporting Information 2** PRISMA 2020 checklist.

## Data Availability

Data supporting the findings of this study are available from the corresponding author upon reasonable request.
