# Supplementary material for: Effects of Nurse‐Led Interventions on Adherence and Clinical Outcomes Among Adults Receiving Noninvasive Ventilation: A Systematic Review of Randomized Controlled Trials
Source: Nurs Res Pract. 2026 Oct 12;2026:7876955. doi: 10.1155/nrp/7876955 (PMC13655774; doi:10.1155/nrp/7876955)
Supplement: Supplementary file 2 [file NRP-2026-7876955-s002.docx]

Pubmed: 213

(((("Adult"[Mesh] OR "Young Adult"[Mesh] OR "Aged"[Mesh] OR "Middle Aged"[Mesh] OR "Aged, 80 and over"[Mesh])) OR (("Adult*"[Title/Abstract] OR "Young Adult*"[Title/Abstract] OR "Adult*, Young"[Title/Abstract] OR "Aged"[Title/Abstract] OR "Elderly"[Title/Abstract] OR "Middle Age*"[Title/Abstract] OR "Aged, 80 and over"[Title/Abstract] OR "Oldest Old"[Title/Abstract]))) AND ((("Noninvasive Ventilation"[Mesh] OR "Continuous Positive Airway Pressure"[Mesh]) OR (("Noninvasive Ventilation*"[Title/Abstract] OR "Non Invasive Ventilation*"[Title/Abstract] OR "Non-Invasive Ventilation*"[Title/Abstract] OR "Continuous Positive Airway Pressure"[Title/Abstract] OR "CPAP Ventilation"[Title/Abstract] OR "Airway Pressure Release Ventilation"[Title/Abstract] OR "APRV Ventilation Mode*"[Title/Abstract] OR "Nasal Continuous Positive Airway Pressure"[Title/Abstract] OR "nCPAP Ventilation"[Title/Abstract] OR "Biphasic Continuous Positive Airway Pressure"[Title/Abstract] OR "Bilevel Continuous Positive Airway Pressure"[Title/Abstract] OR "Bilevel Positive Airway Pressure"[Title/Abstract] OR "BiPAP"[Title/Abstract] OR "Biphasic Positive Airway Pressure"[Title/Abstract] OR "Respiratory failure"[Title/Abstract] OR "Noninvasive ventilation"[Title/Abstract] OR "NIV"[Title/Abstract] OR "NPPV"[Title/Abstract] OR "CPAP"[Title/Abstract] OR "BiPAP"[Title/Abstract])))) AND (("Psychosocial Intervention"[Mesh] OR "Education"[Mesh] OR "Patient Education as Topic"[Mesh] OR "Health Education"[Mesh] OR "Patient Comfort"[Mesh] OR "Information Motivation Behavioral Skills Model"[Mesh] OR "Patient Positioning"[Mesh] OR "Resilience, Psychological"[Mesh] OR "Feedback, Psychological"[Mesh] OR "Psychological Well-Being"[Mesh] OR "Psychosocial Support Systems"[Mesh]) OR ("Intervention, Psychosocial"[Title/Abstract] OR "Interventions, Psychosocial"[Title/Abstract] OR "Psychosocial Interventions"[Title/Abstract] OR "Psychological Intervention"[Title/Abstract] OR "Intervention, Psychological"[Title/Abstract] OR "Interventions, Psychological"[Title/Abstract] OR "Psychological Interventions"[Title/Abstract] OR "Educational Activities"[Title/Abstract] OR "Activities, Educational"[Title/Abstract] OR "Activity, Educational"[Title/Abstract] OR "Educational Activity"[Title/Abstract] OR "Literacy Programs"[Title/Abstract] OR "Literacy Program"[Title/Abstract] OR "Program, Literacy"[Title/Abstract] OR "Programs, Literacy"[Title/Abstract] OR "Training Programs"[Title/Abstract] OR "Programs, Training"[Title/Abstract] OR "Program, Training"[Title/Abstract] OR "Training Program"[Title/Abstract] OR "Workshops"[Title/Abstract] OR "Workshop"[Title/Abstract] OR "curriculum"[Title/Abstract] OR "teaching"[Title/Abstract] OR "training"[Title/Abstract] OR "Education of Patients"[Title/Abstract] OR "Education, Patient"[Title/Abstract] OR "Patient Education"[Title/Abstract] OR "Education, Health"[Title/Abstract] OR "Community Health Education"[Title/Abstract] OR "Education, Community Health"[Title/Abstract] OR "Health Education, Community"[Title/Abstract] OR "Comfort, Patient"[Title/Abstract] OR "Comfort Care"[Title/Abstract] OR "Care, Comfort"[Title/Abstract] OR "Information-Motivation-Behavioral Skills Model"[Title/Abstract] OR "Model, Information-Motivation-Behavioral Skills"[Title/Abstract] OR "Patient Positionings"[Title/Abstract] OR "Positioning, Patient"[Title/Abstract] OR "Psychological Resilience"[Title/Abstract] OR "Resiliency, Psychological"[Title/Abstract] OR "Psychological Resiliency"[Title/Abstract] OR "Resilience"[Title/Abstract] OR "Resiliences"[Title/Abstract] OR "Resiliency"[Title/Abstract] OR "Resiliencies"[Title/Abstract] OR "Stress Immunity"[Title/Abstract] OR "Psychological Feedback"[Title/Abstract] OR "Psychological Well Being"[Title/Abstract] OR "Well-Being, Psychological"[Title/Abstract] OR "Psychological Wellness"[Title/Abstract] OR "Wellness, Psychological"[Title/Abstract] OR "Psychological Ill-Being"[Title/Abstract] OR "Ill-Being, Psychological"[Title/Abstract] OR "Psychological Ill Being"[Title/Abstract] OR "Psychosocial Support System"[Title/Abstract] OR "Support System, Psychosocial"[Title/Abstract] OR "Support Systems, Psychosocial"[Title/Abstract] OR "Psychological Support System"[Title/Abstract] OR "Psychological Support Systems"[Title/Abstract] OR "Support System, Psychological"[Title/Abstract] OR "Support Systems, Psychological"[Title/Abstract] OR "Social Support System"[Title/Abstract] OR "Social Support Systems"[Title/Abstract] OR "Support Systems, Social"[Title/Abstract] OR "Systems, Social Support"[Title/Abstract] OR "Psychosocial Support"[Title/Abstract] OR "Psychosocial Supports"[Title/Abstract] OR "Support, Psychosocial"[Title/Abstract] OR "Supports, Psychosocial"[Title/Abstract] OR "Nursing Care"[Title/Abstract] OR "Nursing Intervention"[Title/Abstract] OR "Patient Care Planning"[Title/Abstract] OR "Nursing Process"[Title/Abstract] OR "Advanced Practice Nursing"[Title/Abstract] OR "Evidence-Based Nursing"[Title/Abstract] OR "nursing intervention"[Title/Abstract] OR "nursing interventions"[Title/Abstract] OR "nurse-led intervention"[Title/Abstract] OR "nurse-led interventions"[Title/Abstract] OR "nursing care intervention"[Title/Abstract] OR "nursing management"[Title/Abstract] OR "nurse-led care"[Title/Abstract] OR "nurse-driven intervention"[Title/Abstract] OR "nurse-initiated intervention"[Title/Abstract] OR "nurse-managed care"[Title/Abstract] OR "APN intervention"[Title/Abstract] OR "advanced practice nurse intervention"[Title/Abstract]))) AND ((("Treatment Adherence and Compliance"[Mesh] OR "Patient Compliance"[Mesh] OR "Guideline Adherence"[Mesh]) OR "Duration of Therapy"[Mesh]) OR ("Treatment Adherence and Compliance"[Title/Abstract] OR "Patient Compliance"[Title/Abstract] OR "Guideline Adherence"[Title/Abstract] OR "Duration of Therapy"[Title/Abstract] OR "Therapy Duration"[Title/Abstract] OR "Duration of Treatment"[Title/Abstract] OR "Treatment Duration"[Title/Abstract] OR "Therapeutic Adherence and Compliance"[Title/Abstract] OR "Treatment Adherence"[Title/Abstract] OR "Adherence, Treatment"[Title/Abstract] OR "Therapeutic Adherence"[Title/Abstract] OR "Adherence, Therapeutic"[Title/Abstract] OR "Compliance, Patient"[Title/Abstract] OR "Client Compliance"[Title/Abstract] OR "Compliance, Client"[Title/Abstract] OR "Client Adherence"[Title/Abstract] OR "Adherence, Client"[Title/Abstract] OR "Patient Cooperation"[Title/Abstract] OR "Cooperation, Patient"[Title/Abstract] OR "Patient Adherence"[Title/Abstract] OR "Adherence, Patient"[Title/Abstract] OR "Patient Non-Compliance"[Title/Abstract] OR "Non-Compliance, Patient"[Title/Abstract] OR "Patient Non Compliance"[Title/Abstract] OR "Non-Adherent Patient"[Title/Abstract] OR "Non Adherent Patient"[Title/Abstract] OR "Non-Adherent Patients"[Title/Abstract] OR "Patient, Non-Adherent"[Title/Abstract] OR "Patient Non-Adherence"[Title/Abstract] OR "Non-Adherence, Patient"[Title/Abstract] OR "Patient Non Adherence"[Title/Abstract] OR "Patient Nonadherence"[Title/Abstract] OR "Patient Noncompliance"[Title/Abstract] OR "Treatment Compliance"[Title/Abstract] OR "Compliance, Treatment"[Title/Abstract] OR "Treatment Compliances"[Title/Abstract] OR "Therapeutic Compliance"[Title/Abstract] OR "Compliance, Therapeutic"[Title/Abstract] OR "Therapeutic Compliances"[Title/Abstract] OR "Adherence, Guideline"[Title/Abstract] OR "Institutional Adherence"[Title/Abstract] OR "Policy Compliance"[Title/Abstract] OR "Compliance, Policy"[Title/Abstract] OR "Protocol Compliance"[Title/Abstract] OR "Compliance, Protocol"[Title/Abstract] OR "tolerability"[Title/Abstract] OR "treatment tolerability"[Title/Abstract] OR "therapy tolerability"[Title/Abstract] OR "tolerance"[Title/Abstract] OR "treatment tolerance"[Title/Abstract] OR "treatment intolerance"[Title/Abstract] OR "safety and tolerability"[Title/Abstract] OR "patient-reported tolerability"[Title/Abstract] OR "acceptability"[Title/Abstract]))

Scopus: 490

( ( TITLE-ABS-KEY ( ( "Adult" Mesh OR "Young Adult" Mesh OR "Aged" Mesh OR "Middle Aged" Mesh OR "Aged, 80 and over" Mesh ) ) OR TITLE-ABS-KEY ( ( "Adult*" OR "Young Adult*" OR "Adult*, Young" OR "Aged" OR "Elderly" OR "Middle Age*" OR "Aged, 80 and over" OR "Oldest Old" ) ) ) ) AND ( ( TITLE-ABS-KEY ( ( "Noninvasive Ventilation" Mesh OR "Continuous Positive Airway Pressure" Mesh ) ) OR TITLE-ABS-KEY ( ( "Noninvasive Ventilation*" OR "Non Invasive Ventilation*" OR "Non-Invasive Ventilation*" OR "Continuous Positive Airway Pressure" OR "CPAP Ventilation" OR "Airway Pressure Release Ventilation" OR "APRV Ventilation Mode*" OR "Nasal Continuous Positive Airway Pressure" OR "nCPAP Ventilation" OR "Biphasic Continuous Positive Airway Pressure" OR "Bilevel Continuous Positive Airway Pressure" OR "Bilevel Positive Airway Pressure" OR "BiPAP" OR "Biphasic Positive Airway Pressure" OR "Respiratory failure" OR "Noninvasive ventilation" OR "NIV" OR "NPPV" OR "CPAP" OR "BiPAP" ) ) ) ) AND ( ( TITLE-ABS-KEY ( "Psychosocial Intervention" Mesh OR "Education" Mesh OR "Patient Education as Topic" Mesh OR "Health Education" Mesh OR "Patient Comfort" Mesh OR "Information Motivation Behavioral Skills Model" Mesh OR "Patient Positioning" Mesh OR "Resilience, Psychological" Mesh OR "Feedback, Psychological" Mesh OR "Psychological Well-Being" Mesh OR "Psychosocial Support Systems" Mesh ) OR TITLE-ABS-KEY ( "Intervention, Psychosocial" OR "Interventions, Psychosocial" OR "Psychosocial Interventions" OR "Psychological Intervention" OR "Intervention, Psychological" OR "Interventions, Psychological" OR "Psychological Interventions" OR "Educational Activities" OR "Activities, Educational" OR "Activity, Educational" OR "Educational Activity" OR "Literacy Programs" OR "Literacy Program" OR "Program, Literacy" OR "Programs, Literacy" OR "Training Programs" OR "Programs, Training" OR "Program, Training" OR "Training Program" OR "Workshops" OR "Workshop" OR "curriculum" OR "teaching" OR "training" OR "Education of Patients" OR "Education, Patient" OR "Patient Education" OR "Education, Health" OR "Community Health Education" OR "Education, Community Health" OR "Health Education, Community" OR "Comfort, Patient" OR "Comfort Care" OR "Care, Comfort" OR "Information-Motivation-Behavioral Skills Model" OR "Model, Information-Motivation-Behavioral Skills" OR "Patient Positionings" OR "Positioning, Patient" OR "Psychological Resilience" OR "Resiliency, Psychological" OR "Psychological Resiliency" OR "Resilience" OR "Resiliences" OR "Resiliency" OR "Resiliencies" OR "Stress Immunity" OR "Psychological Feedback" OR "Psychological Well Being" OR "Well-Being, Psychological" OR "Psychological Wellness" OR "Wellness, Psychological" OR "Psychological Ill-Being" OR "Ill-Being, Psychological" OR "Psychological Ill Being" OR "Psychosocial Support System" OR "Support System, Psychosocial" OR "Support Systems, Psychosocial" OR "Psychological Support System" OR "Psychological Support Systems" OR "Support System, Psychological" OR "Support Systems, Psychological" OR "Social Support System" OR "Social Support Systems" OR "Support Systems, Social" OR "Systems, Social Support" OR "Psychosocial Support" OR "Psychosocial Supports" OR "Support, Psychosocial" OR "Supports, Psychosocial" OR "Nursing Care" OR "Nursing Intervention" OR "Patient Care Planning" OR "Nursing Process" OR "Advanced Practice Nursing" OR "Evidence-Based Nursing" OR "nursing intervention" OR "nursing interventions" OR "nurse-led intervention" OR "nurse-led interventions" OR "nursing care intervention" OR "nursing management" OR "nurse-led care" OR "nurse-driven intervention" OR "nurse-initiated intervention" OR "nurse-managed care" OR "APN intervention" OR "advanced practice nurse intervention" ) ) ) AND ( ( TITLE-ABS-KEY ( ( "Treatment Adherence and Compliance" Mesh OR "Patient Compliance" Mesh OR "Guideline Adherence" Mesh ) OR "Duration of Therapy" Mesh ) OR TITLE-ABS-KEY ( "Treatment Adherence and Compliance" OR "Patient Compliance" OR "Guideline Adherence" OR "Duration of Therapy" OR "Therapy Duration" OR "Duration of Treatment" OR "Treatment Duration" OR "Therapeutic Adherence and Compliance" OR "Treatment Adherence" OR "Adherence, Treatment" OR "Therapeutic Adherence" OR "Adherence, Therapeutic" OR "Compliance, Patient" OR "Client Compliance" OR "Compliance, Client" OR "Client Adherence" OR "Adherence, Client" OR "Patient Cooperation" OR "Cooperation, Patient" OR "Patient Adherence" OR "Adherence, Patient" OR "Patient Non-Compliance" OR "Non-Compliance, Patient" OR "Patient Non Compliance" OR "Non-Adherent Patient" OR "Non Adherent Patient" OR "Non-Adherent Patients" OR "Patient, Non-Adherent" OR "Patient Non-Adherence" OR "Non-Adherence, Patient" OR "Patient Non Adherence" OR "Patient Nonadherence" OR "Patient Noncompliance" OR "Treatment Compliance" OR "Compliance, Treatment" OR "Treatment Compliances" OR "Therapeutic Compliance" OR "Compliance, Therapeutic" OR "Therapeutic Compliances" OR "Adherence, Guideline" OR "Institutional Adherence" OR "Policy Compliance" OR "Compliance, Policy" OR "Protocol Compliance" OR "Compliance, Protocol" OR "tolerability" OR "treatment tolerability" OR "therapy tolerability" OR "tolerance" OR "treatment tolerance" OR "treatment intolerance" OR "safety and tolerability" OR "patient-reported tolerability" OR "acceptability" ) ) )

Cochrane: 285

#1 "Adults" OR "Adults, Young" OR "Adult, Young" OR "Young Adults" OR "Elderly" OR "Middle Age" OR "Oldest Old" OR "Adult" OR "Young Adult" OR "Aged" OR "Middle Aged" OR "Aged, 80 and over" 1409006

#2 "Noninvasive Ventilations" OR "Ventilation, Noninvasive" OR "Ventilations, Noninvasive" OR "Non Invasive Ventilation" OR "Non Invasive Ventilations" OR "Ventilation, Non Invasive" OR "Ventilations, Non Invasive" OR "Non-Invasive Ventilation" OR "Non-Invasive Ventilations" OR "Ventilation, Non-Invasive" OR "Ventilations, Non-Invasive" OR "CPAP Ventilation" OR "Ventilation, CPAP" OR "Airway Pressure Release Ventilation" OR "APRV Ventilation Mode" OR "APRV Ventilation Modes" OR "Ventilation Mode, APRV" OR "Ventilation Modes, APRV" OR "Nasal Continuous Positive Airway Pressure" OR "nCPAP Ventilation" OR "Ventilation, nCPAP" OR "Biphasic Continuous Positive Airway Pressure" OR "Bilevel Continuous Positive Airway Pressure" OR "Bilevel Positive Airway Pressure" OR "BiPAP Bilevel Positive Airway Pressure" OR "Biphasic Positive Airway Pressure" OR "BiPAP Biphasic Positive Airway Pressure" OR "Noninvasive Ventilation" OR "Continuous Positive Airway Pressure" 8772

#3 "Intervention, Psychosocial" OR "Interventions, Psychosocial" OR "Psychosocial Interventions" OR "Psychological Intervention" OR "Intervention, Psychological" OR "Interventions, Psychological" OR "Psychological Interventions" OR "Educational Activities" OR "Activities, Educational" OR "Activity, Educational" OR "Educational Activity" OR "Literacy Programs" OR "Literacy Program" OR "Program, Literacy" OR "Programs, Literacy" OR "Training Programs" OR "Programs, Training" OR "Program, Training" OR "Training Program" OR "Workshops" OR "Workshop" OR "curriculum" OR "teaching" OR "training" OR "Education of Patients" OR "Education, Patient" OR "Patient Education" OR "Education, Health" OR "Community Health Education" OR "Education, Community Health" OR "Health Education, Community" OR "Comfort, Patient" OR "Comfort Care" OR "Care, Comfort" OR "Information-Motivation-Behavioral Skills Model" OR "Information-Motivation-Behavioral Skills Models" OR "Model, Information-Motivation-Behavioral Skills" OR "Models, Information-Motivation-Behavioral Skills" OR "Skills Model, Information-Motivation-Behavioral" OR "Skills Models, Information-Motivation-Behavioral" OR "Patient Positionings" OR "Positioning, Patient" OR "Positionings, Patient" OR "Psychological Resilience" OR "Resiliency, Psychological" OR "Psychological Resiliency" OR "Resilience" OR "Resiliences" OR "Resiliency" OR "Resiliencies" OR "Resilient Responses, Psychological" OR "Psychological Resilient Response" OR "Resilient Response, Psychological" OR "Response, Psychological Resilient" OR "Psychological Resilient Responses" OR "Stress Immunity" OR "Immunity, Stress" OR "Stress Immunities" OR "Feedbacks, Psychological" OR "Psychological Feedbacks" OR "Psychological Feedback" OR "Psychological Well Being" OR "Well-Being, Psychological" OR "Psychological Wellness" OR "Wellness, Psychological" OR "Psychological Ill-Being" OR "Ill-Being, Psychological" OR "Psychological Ill Being" OR "Psychosocial Support System" OR "Support System, Psychosocial" OR "Support Systems, Psychosocial" OR "Psychological Support System" OR "Psychological Support Systems" OR "Support System, Psychological" OR "Support Systems, Psychological" OR "System, Psychological Support" OR "Systems, Psychological Support" OR "Social Support System" OR "Social Support Systems" OR "Support System, Social" OR "Support Systems, Social" OR "System, Social Support" OR "Systems, Social Support" OR "Psychosocial Support" OR "Psychosocial Supports" OR "Support, Psychosocial" OR "Supports, Psychosocial" OR "Psychosocial Intervention" OR "Education" OR "education [Subheading]" OR "Patient Education as Topic" OR "Health Education" OR "Patient Comfort" OR "Information Motivation Behavioral Skills Model" OR "Patient Positioning" OR "Resilience, Psychological" OR "Feedback, Psychological" OR "Psychological Well-Being" OR "Psychosocial Support Systems" OR "Nursing Care" OR "Nursing Intervention" OR "Patient Care Planning" OR "Nursing Process" OR "Advanced Practice Nursing" OR "Evidence-Based Nursing" OR "nursing intervention" OR "nursing interventions" OR "nurse-led intervention" OR "nurse-led interventions" OR "nursing care intervention" OR "nursing management" OR "nurse-led care" OR "nurse-driven intervention" OR "nurse-initiated intervention" OR "nurse-managed care" OR "APN intervention" OR "advanced practice nurse intervention" 296766

#4 "Therapeutic Adherence and Compliance" OR "Treatment Adherence" OR "Adherence, Treatment" OR "Therapeutic Adherence" OR "Adherence, Therapeutic" OR "Compliance, Patient" OR "Client Compliance" OR "Client Compliances" OR "Compliance, Client" OR "Client Adherence" OR "Adherence, Client" OR "Patient Cooperation" OR "Cooperation, Patient" OR "Patient Adherence" OR "Adherence, Patient" OR "Patient Non-Compliance" OR "Non-Compliance, Patient" OR "Patient Non Compliance" OR "Non-Adherent Patient" OR "Non Adherent Patient" OR "Non-Adherent Patients" OR "Patient, Non-Adherent" OR "Patient Non-Adherence" OR "Non-Adherence, Patient" OR "Patient Non Adherence" OR "Patient Nonadherence" OR "Nonadherence, Patient" OR "Patient Noncompliance" OR "Noncompliance, Patient" OR "Treatment Compliance" OR "Compliance, Treatment" OR "Treatment Compliances" OR "Therapeutic Compliance" OR "Compliance, Therapeutic" OR "Therapeutic Compliances" OR "Adherence, Guideline" OR "Institutional Adherence" OR "Adherence, Institutional" OR "Policy Compliance" OR "Compliance, Policy" OR "Protocol Compliance" OR "Compliance, Protocol" OR "Treatment Adherence and Compliance" OR "Patient Compliance" OR "Guideline Adherence" OR "tolerability" OR "treatment tolerability" OR "therapy tolerability" OR "tolerance" OR "treatment tolerance" OR "treatment intolerance" OR "safety and tolerability" OR "patient-reported tolerability" OR "acceptability" 192835

#5 #1 AND #2 AND #3 AND #4 285

Embase: 582

#1 "Adults" OR "Adults, Young" OR "Adult, Young" OR "Young Adults" OR "Elderly" OR "Middle Age" OR "Oldest Old" OR "Adult" OR "Young Adult" OR "Aged" OR "Middle Aged" OR "Aged, 80 and over" 14872699

#2 "Noninvasive Ventilations" OR "Ventilation, Noninvasive" OR "Ventilations, Noninvasive" OR "Non Invasive Ventilation" OR "Non Invasive Ventilations" OR "Ventilation, Non Invasive" OR "Ventilations, Non Invasive" OR "Non-Invasive Ventilation" OR "Non-Invasive Ventilations" OR "Ventilation, Non-Invasive" OR "Ventilations, Non-Invasive" OR "CPAP Ventilation" OR "Ventilation, CPAP" OR "Airway Pressure Release Ventilation" OR "APRV Ventilation Mode" OR "APRV Ventilation Modes" OR "Ventilation Mode, APRV" OR "Ventilation Modes, APRV" OR "Nasal Continuous Positive Airway Pressure" OR "nCPAP Ventilation" OR "Ventilation, nCPAP" OR "Biphasic Continuous Positive Airway Pressure" OR "Bilevel Continuous Positive Airway Pressure" OR "Bilevel Positive Airway Pressure" OR "BiPAP Bilevel Positive Airway Pressure" OR "Biphasic Positive Airway Pressure" OR "BiPAP Biphasic Positive Airway Pressure" OR "Noninvasive Ventilation" OR "Continuous Positive Airway Pressure" 63748

#3 "Intervention, Psychosocial" OR "Interventions, Psychosocial" OR "Psychosocial Interventions" OR "Psychological Intervention" OR "Intervention, Psychological" OR "Interventions, Psychological" OR "Psychological Interventions" OR "Educational Activities" OR "Activities, Educational" OR "Activity, Educational" OR "Educational Activity" OR "Literacy Programs" OR "Literacy Program" OR "Program, Literacy" OR "Programs, Literacy" OR "Training Programs" OR "Programs, Training" OR "Program, Training" OR "Training Program" OR "Workshops" OR "Workshop" OR "curriculum" OR "teaching" OR "training" OR "Education of Patients" OR "Education, Patient" OR "Patient Education" OR "Education, Health" OR "Community Health Education" OR "Education, Community Health" OR "Health Education, Community" OR "Comfort, Patient" OR "Comfort Care" OR "Care, Comfort" OR "Information-Motivation-Behavioral Skills Model" OR "Information-Motivation-Behavioral Skills Models" OR "Model, Information-Motivation-Behavioral Skills" OR "Models, Information-Motivation-Behavioral Skills" OR "Skills Model, Information-Motivation-Behavioral" OR "Skills Models, Information-Motivation-Behavioral" OR "Patient Positionings" OR "Positioning, Patient" OR "Positionings, Patient" OR "Psychological Resilience" OR "Resiliency, Psychological" OR "Psychological Resiliency" OR "Resilience" OR "Resiliences" OR "Resiliency" OR "Resiliencies" OR "Resilient Responses, Psychological" OR "Psychological Resilient Response" OR "Resilient Response, Psychological" OR "Response, Psychological Resilient" OR "Psychological Resilient Responses" OR "Stress Immunity" OR "Immunity, Stress" OR "Stress Immunities" OR "Feedbacks, Psychological" OR "Psychological Feedbacks" OR "Psychological Feedback" OR "Psychological Well Being" OR "Well-Being, Psychological" OR "Psychological Wellness" OR "Wellness, Psychological" OR "Psychological Ill-Being" OR "Ill-Being, Psychological" OR "Psychological Ill Being" OR "Psychosocial Support System" OR "Support System, Psychosocial" OR "Support Systems, Psychosocial" OR "Psychological Support System" OR "Psychological Support Systems" OR "Support System, Psychological" OR "Support Systems, Psychological" OR "System, Psychological Support" OR "Systems, Psychological Support" OR "Social Support System" OR "Social Support Systems" OR "Support System, Social" OR "Support Systems, Social" OR "System, Social Support" OR "Systems, Social Support" OR "Psychosocial Support" OR "Psychosocial Supports" OR "Support, Psychosocial" OR "Supports, Psychosocial" OR "Psychosocial Intervention" OR "Education" OR "education [Subheading]" OR "Patient Education as Topic" OR "Health Education" OR "Patient Comfort" OR "Information Motivation Behavioral Skills Model" OR "Patient Positioning" OR "Resilience, Psychological" OR "Feedback, Psychological" OR "Psychological Well-Being" OR "Psychosocial Support Systems" OR "Nursing Care" OR "Nursing Intervention" OR "Patient Care Planning" OR "Nursing Process" OR "Advanced Practice Nursing" OR "Evidence-Based Nursing" OR "nursing intervention" OR "nursing interventions" OR "nurse-led intervention" OR "nurse-led interventions" OR "nursing care intervention" OR "nursing management" OR "nurse-led care" OR "nurse-driven intervention" OR "nurse-initiated intervention" OR "nurse-managed care" OR "APN intervention" OR "advanced practice nurse intervention" 4375140

#4 "Therapeutic Adherence and Compliance" OR "Treatment Adherence" OR "Adherence, Treatment" OR "Therapeutic Adherence" OR "Adherence, Therapeutic" OR "Compliance, Patient" OR "Client Compliance" OR "Client Compliances" OR "Compliance, Client" OR "Client Adherence" OR "Adherence, Client" OR "Patient Cooperation" OR "Cooperation, Patient" OR "Patient Adherence" OR "Adherence, Patient" OR "Patient Non-Compliance" OR "Non-Compliance, Patient" OR "Patient Non Compliance" OR "Non-Adherent Patient" OR "Non Adherent Patient" OR "Non-Adherent Patients" OR "Patient, Non-Adherent" OR "Patient Non-Adherence" OR "Non-Adherence, Patient" OR "Patient Non Adherence" OR "Patient Nonadherence" OR "Nonadherence, Patient" OR "Patient Noncompliance" OR "Noncompliance, Patient" OR "Treatment Compliance" OR "Compliance, Treatment" OR "Treatment Compliances" OR "Therapeutic Compliance" OR "Compliance, Therapeutic" OR "Therapeutic Compliances" OR "Adherence, Guideline" OR "Institutional Adherence" OR "Adherence, Institutional" OR "Policy Compliance" OR "Compliance, Policy" OR "Protocol Compliance" OR "Compliance, Protocol" OR "Treatment Adherence and Compliance" OR "Patient Compliance" OR "Guideline Adherence" OR "tolerability" OR "treatment tolerability" OR "therapy tolerability" OR "tolerance" OR "treatment tolerance" OR "treatment intolerance" OR "safety and tolerability" OR "patient-reported tolerability" OR "acceptability" 1199453

#5 #1 AND #2 AND #3 AND #4 582

Web of science: 45

#1 ("Adult"[Mesh] OR "Young Adult"[Mesh] OR "Aged"[Mesh] OR "Middle Aged"[Mesh] OR "Aged, 80 and over"[Mesh]) (Topic) or ("Adult*" OR "Young Adult*" OR "Adult*, Young" OR "Aged" OR "Elderly" OR "Middle Age*" OR "Aged, 80 and over" OR "Oldest Old") (Topic) 3498342

#2 ("Noninvasive Ventilation"[Mesh] OR "Continuous Positive Airway Pressure"[Mesh]) (Topic) or ("Noninvasive Ventilation*" OR "Non Invasive Ventilation*" OR "Non-Invasive Ventilation*" OR "Continuous Positive Airway Pressure" OR "CPAP Ventilation" OR "Airway Pressure Release Ventilation" OR "APRV Ventilation Mode*" OR "Nasal Continuous Positive Airway Pressure" OR "nCPAP Ventilation" OR "Biphasic Continuous Positive Airway Pressure" OR "Bilevel Continuous Positive Airway Pressure" OR "Bilevel Positive Airway Pressure" OR "BiPAP" OR "Biphasic Positive Airway Pressure" OR "Respiratory failure" OR "Noninvasive ventilation" OR "NIV" OR "NPPV" OR "CPAP" OR "BiPAP") 81388

#3 "Psychosocial Intervention"[Mesh] OR "Education"[Mesh] OR "Patient Education as Topic"[Mesh] OR "Health Education"[Mesh] OR "Patient Comfort"[Mesh] OR "Information Motivation Behavioral Skills Model"[Mesh] OR "Patient Positioning"[Mesh] OR "Resilience, Psychological"[Mesh] OR "Feedback, Psychological"[Mesh] OR "Psychological Well-Being"[Mesh] OR "Psychosocial Support Systems"[Mesh] (Topic) or "Intervention, Psychosocial" OR "Interventions, Psychosocial" OR "Psychosocial Interventions" OR "Psychological Intervention" OR "Intervention, Psychological" OR "Interventions, Psychological" OR "Psychological Interventions" OR "Educational Activities" OR "Activities, Educational" OR "Activity, Educational" OR "Educational Activity" OR "Literacy Programs" OR "Literacy Program" OR "Program, Literacy" OR "Programs, Literacy" OR "Training Programs" OR "Programs, Training" OR "Program, Training" OR "Training Program" OR "Workshops" OR "Workshop" OR "curriculum" OR "teaching" OR "training" OR "Education of Patients" OR "Education, Patient" OR "Patient Education" OR "Education, Health" OR "Community Health Education" OR "Education, Community Health" OR "Health Education, Community" OR "Comfort, Patient" OR "Comfort Care" OR "Care, Comfort" OR "Information-Motivation-Behavioral Skills Model" OR "Model, Information-Motivation-Behavioral Skills" OR "Patient Positionings" OR "Positioning, Patient" OR "Psychological Resilience" OR "Resiliency, Psychological" OR "Psychological Resiliency" OR "Resilience" OR "Resiliences" OR "Resiliency" OR "Resiliencies" OR "Stress Immunity" OR "Psychological Feedback" OR "Psychological Well Being" OR "Well-Being, Psychological" OR "Psychological Wellness" OR "Wellness, Psychological" OR "Psychological Ill-Being" OR "Ill-Being, Psychological" OR "Psychological Ill Being" OR "Psychosocial Support System" OR "Support System, Psychosocial" OR "Support Systems, Psychosocial" OR "Psychological Support System" OR "Psychological Support Systems" OR "Support System, Psychological" OR "Support Systems, Psychological" OR "Social Support System" OR "Social Support Systems" OR "Support Systems, Social" OR "Systems, Social Support" OR "Psychosocial Support" OR "Psychosocial Supports" OR "Support, Psychosocial" OR "Supports, Psychosocial" OR "Nursing Care" OR "Nursing Intervention" OR "Patient Care Planning" OR "Nursing Process" OR "Advanced Practice Nursing" OR "Evidence-Based Nursing" OR "nursing intervention" OR "nursing interventions" OR "nurse-led intervention" OR "nurse-led interventions" OR "nursing care intervention" OR "nursing management" OR "nurse-led care" OR "nurse-driven intervention" OR "nurse-initiated intervention" OR "nurse-managed care" OR "APN intervention" OR "advanced practice nurse intervention" 2553305

#4 ("Treatment Adherence and Compliance"[Mesh] OR "Patient Compliance"[Mesh] OR "Guideline Adherence"[Mesh]) OR "Duration of Therapy"[Mesh] (Topic) or "Treatment Adherence and Compliance" OR "Patient Compliance" OR "Guideline Adherence" OR "Duration of Therapy" OR "Therapy Duration" OR "Duration of Treatment" OR "Treatment Duration" OR "Therapeutic Adherence and Compliance" OR "Treatment Adherence" OR "Adherence, Treatment" OR "Therapeutic Adherence" OR "Adherence, Therapeutic" OR "Compliance, Patient" OR "Client Compliance" OR "Compliance, Client" OR "Client Adherence" OR "Adherence, Client" OR "Patient Cooperation" OR "Cooperation, Patient" OR "Patient Adherence" OR "Adherence, Patient" OR "Patient Non-Compliance" OR "Non-Compliance, Patient" OR "Patient Non Compliance" OR "Non-Adherent Patient" OR "Non Adherent Patient" OR "Non-Adherent Patients" OR "Patient, Non-Adherent" OR "Patient Non-Adherence" OR "Non-Adherence, Patient" OR "Patient Non Adherence" OR "Patient Nonadherence" OR "Patient Noncompliance" OR "Treatment Compliance" OR "Compliance, Treatment" OR "Treatment Compliances" OR "Therapeutic Compliance" OR "Compliance, Therapeutic" OR "Therapeutic Compliances" OR "Adherence, Guideline" OR "Institutional Adherence" OR "Policy Compliance" OR "Compliance, Policy" OR "Protocol Compliance" OR "Compliance, Protocol" OR "tolerability" OR "treatment tolerability" OR "therapy tolerability" OR "tolerance" OR "treatment tolerance" OR "treatment intolerance" OR "safety and tolerability" OR "patient-reported tolerability" OR "acceptability" (Topic) 829902

#5 #1 AND #2 AND #3 AND #4 45

CINAHL: 5

AB ("Adults" OR "Adults, Young" OR "Adult, Young" OR "Young Adults" OR "Elderly" OR "Middle Age" OR "Oldest Old" OR "Adult" OR "Young Adult" OR "Aged" OR "Middle Aged" OR "Aged, 80 and over") AND AB ("Noninvasive Ventilations" OR"Ventilation, Noninvasive" OR"Ventilations, Noninvasive" OR"Non Invasive Ventilation" OR"Non Invasive Ventilations" OR"Ventilation, Non Invasive" OR"Ventilations, Non Invasive" OR"Non-Invasive Ventilation" OR"Non-Invasive Ventilations" OR"Ventilation, Non-Invasive" OR"Ventilations, Non-Invasive" OR"CPAP Ventilation" OR"Ventilation, CPAP" OR"Airway Pressure Release Ventilation" OR"APRV Ventilation Mode" OR"APRV Ventilation Modes" OR"Ventilation Mode, APRV" OR"Ventilation Modes, APRV" OR"Nasal Continuous Positive Airway Pressure" OR"nCPAP Ventilation" OR"Ventilation, nCPAP" OR"Biphasic Continuous Positive Airway Pressure" OR"Bilevel Continuous Positive Airway Pressure" OR"Bilevel Positive Airway Pressure" OR"BiPAP Bilevel Positive Airway Pressure" OR"Biphasic Positive Airway Pressure" OR"BiPAP Biphasic Positive Airway Pressure" OR "Noninvasive Ventilation" OR"Continuous Positive Airway Pressure") AND AB ("Intervention, Psychosocial" OR"Interventions, Psychosocial" OR"Psychosocial Interventions" OR"Psychological Intervention" OR"Intervention, Psychological" OR"Interventions, Psychological" OR"Psychological Interventions" OR"Educational Activities" OR"Activities, Educational" OR"Activity, Educational" OR"Educational Activity" OR"Literacy Programs" OR"Literacy Program" OR"Program, Literacy" OR"Programs, Literacy" OR"Training Programs" OR"Programs, Training" OR"Program, Training" OR"Training Program" OR"Workshops" OR"Workshop" OR"curriculum" OR"teaching" OR"training" OR"Education of Patients" OR"Education, Patient" OR"Patient Education" OR"Education, Health" OR"Community Health Education" OR"Education, Community Health" OR"Health Education, Community" OR"Comfort, Patient" OR"Comfort Care" OR"Care, Comfort" OR"Information-Motivation-Behavioral Skills Model" OR"Information-Motivation-Behavioral Skills Models" OR"Model, Information-Motivation-Behavioral Skills" OR"Models, Information-Motivation-Behavioral Skills" OR"Skills Model, Information-Motivation-Behavioral" OR"Skills Models, Information-Motivation-Behavioral" OR"Patient Positionings" OR"Positioning, Patient" OR"Positionings, Patient" OR"Psychological Resilience" OR"Resiliency, Psychological" OR"Psychological Resiliency" OR"Resilience" OR"Resiliences" OR"Resiliency" OR"Resiliencies" OR"Resilient Responses, Psychological" OR"Psychological Resilient Response" OR"Resilient Response, Psychological" OR"Response, Psychological Resilient" OR"Psychological Resilient Responses" OR"Stress Immunity" OR"Immunity, Stress" OR"Stress Immunities" OR"Feedbacks, Psychological" OR"Psychological Feedbacks" OR"Psychological Feedback" OR"Psychological Well Being" OR"Well-Being, Psychological" OR"Psychological Wellness" OR"Wellness, Psychological" OR"Psychological Ill-Being" OR"Ill-Being, Psychological" OR"Psychological Ill Being" OR"Psychosocial Support System" OR"Support System, Psychosocial" OR"Support Systems, Psychosocial" OR"Psychological Support System" OR"Psychological Support Systems" OR"Support System, Psychological" OR"Support Systems, Psychological" OR"System, Psychological Support" OR"Systems, Psychological Support" OR"Social Support System" OR"Social Support Systems" OR"Support System, Social" OR"Support Systems, Social" OR"System, Social Support" OR"Systems, Social Support" OR"Psychosocial Support" OR"Psychosocial Supports" OR"Support, Psychosocial" OR"Supports, Psychosocial" OR"Psychosocial Intervention" OR"Education" OR"education [Subheading]" OR"Patient Education as Topic" OR"Health Education" OR"Patient Comfort" OR"Information Motivation Behavioral Skills Model" OR"Patient Positioning" OR"Resilience, Psychological" OR"Feedback, Psychological" OR"Psychological Well-Being" OR"Psychosocial Support Systems" OR "Nursing Care"OR "Nursing Intervention"OR "Patient Care Planning"OR "Nursing Process"OR "Advanced Practice Nursing"OR "Evidence-Based Nursing"OR "nursing intervention"OR "nursing interventions"OR "nurse-led intervention"OR "nurse-led interventions"OR "nursing care intervention"OR "nursing management"OR "nurse-led care"OR "nurse-driven intervention"OR "nurse-initiated intervention"OR "nurse-managed care"OR "APN intervention"OR "advanced practice nurse intervention") AND AB ("Therapeutic Adherence and Compliance" OR"Treatment Adherence" OR"Adherence, Treatment" OR"Therapeutic Adherence" OR"Adherence, Therapeutic" OR"Compliance, Patient" OR"Client Compliance" OR"Client Compliances" OR"Compliance, Client" OR"Client Adherence" OR"Adherence, Client" OR"Patient Cooperation" OR"Cooperation, Patient" OR"Patient Adherence" OR"Adherence, Patient" OR"Patient Non-Compliance" OR"Non-Compliance, Patient" OR"Patient Non Compliance" OR"Non-Adherent Patient" OR"Non Adherent Patient" OR"Non-Adherent Patients" OR"Patient, Non-Adherent" OR"Patient Non-Adherence" OR"Non-Adherence, Patient" OR"Patient Non Adherence" OR"Patient Nonadherence" OR"Nonadherence, Patient" OR"Patient Noncompliance" OR"Noncompliance, Patient" OR"Treatment Compliance" OR"Compliance, Treatment" OR"Treatment Compliances" OR"Therapeutic Compliance" OR"Compliance, Therapeutic" OR"Therapeutic Compliances" OR"Adherence, Guideline" OR"Institutional Adherence" OR"Adherence, Institutional" OR"Policy Compliance" OR"Compliance, Policy" OR"Protocol Compliance" OR"Compliance, Protocol" OR"Treatment Adherence and Compliance" OR"Patient Compliance" OR"Guideline Adherence" OR "tolerability" OR "treatment tolerability" OR "therapy tolerability" OR "tolerance" OR "treatment tolerance" OR "treatment intolerance" OR "safety and tolerability" OR "patient-reported tolerability" OR "acceptability")
